# Multiple Levels of Heuristic Reasoning Processes in Scientific Model Construction

**DOI:** 10.3389/fpsyg.2022.750713

**Published:** 2022-05-10

**Authors:** John J. Clement

**Affiliations:** Scientific Reasoning Research Institute, College of Education, University of Massachusetts Amherst, Amherst, MA, United States

**Keywords:** reasoning, science, imagery, mental simulation, creativity, heuristics, mental model, grounded cognition

## Abstract

Science historians have recognized the importance of heuristic reasoning strategies for constructing theories, but their extent and degree of organization are still poorly understood. This paper first consolidates a set of important heuristic strategies for constructing scientific models from three books, including studies in the history of genetics and electromagnetism, and an expert think-aloud study in the field of mechanics. The books focus on qualitative reasoning strategies (processes) involved in creative model construction, scientific breakthroughs, and conceptual change. Twenty four processes are examined, most of which are field-general, but all are heuristic in not being guaranteed to work. An organizing framework is then proposed as a four-level hierarchy of nested reasoning processes and subprocesses at different size and time scales, including: Level (L4) Several longer-time-scale *Major Modeling Modes*, such as Model Evolution and Model Competition; the former mode utilizes: (L3) *Modeling Cycle Phases* of Model Generation, Evaluation, and Modification under Constraints*;* which can utilize: (L2) Thirteen *Tactical Heuristic Processes*, e.g., Analogy, Infer new model feature (e.g., by running the model), etc.; many of which selectively utilize: (L1) *Grounded Imagistic Processes*, namely Mental Simulations and Structural Transformations. Incomplete serial ordering in the framework gives it an intermediate degree of organization that is neither anarchistic nor fully algorithmic. Its organizational structure is hypothesized to promote a difficult balance between divergent and convergent processes as it alternates between them in modeling cycles with increasingly constrained modifications. Videotaped think-aloud protocols that include depictive gestures and other imagery indicators indicate that the processes in L1 above can be imagistic. From neurological evidence that imagery uses many of the same brain regions as actual perception and action, it is argued that these expert reasoning processes are grounded in the sense of utilizing the perceptual and motor systems, and interconnections to and possible benefits for reasoning processes at higher levels are examined. The discussion examines whether this grounding and the various forms of organization in the framework may begin to explain how processes that are only sometimes useful and not guaranteed to work can combine successfully to achieve innovative scientific model construction.

## Introduction

This paper is motivated by several long-term questions related to the nature of scientific thinking: What qualitative heuristic reasoning strategies are used by experts during the construction of scientific models? Are these collectively organized in some way? Can any of these expert higher order reasoning strategies be said to be grounded in perceptual or motor processes in some way? In particular, as opposed to large scale strategies for entire research programs (e.g., Lakatos, 1976a), can we understand what medium scale strategies are used by a scientist in constructing models? The work of Polya (1954, 1957) identifying many heuristic strategies in mathematics suggests that there might be a large number in science as well. While these long-term questions have no simple answers, they provide an incentive to assemble and examine a set of examples of strategy use.

This paper begins with an effort to consolidate model construction strategies from three book sources, including one book of my own. Darden (1991) and Nersessian (2008) provide descriptions of reasoning strategies that come from historical case studies of developments in the fields of genetics and electromagnetism, respectively, and they represent two historians of science who approach creative discovery from a deeply informed view of science as problem solving. In Clement (2008) I analyzed think aloud case studies of experts solving explanation problems in mechanics. The books describe difficulties, breakthroughs, conceptual change, and the creative construction of new theories. They focus largely on qualitative modeling, viewed as essential for providing a firm foundation for later quantitative models. Together they describe dozens of scientific reasoning strategies that were used across three different fields of science. Consolidating them will also enable asking the question of whether the strategies are used in random order or are organized in some way.

## Three Detailed Case Studies of Scientific Reasoning

The publication of Darden (1991) identified many more heuristic strategies than previous works on model construction methods in science. It describes the early development of the theory of the gene by multiple scientists. It is unique in assembling and organizing so many strategies within a coherent and detailed history of the development of a scientific theory and can be said to present an image of wide scope.

Nersessian’s (2008) study focuses on James Clerk Maxwell, developer of the modern theory of electromagnetism, culminating in his famous equations. His theory systematizes and explains a huge diversity of electromagnetic phenomena, and is seen on the same level as Newton’s as a synthesis of diverse domains. This book deals with a smaller number of strategies from a single scientist, and this allows it to go into greater depth in making many connections between the development of Maxwell’s theory and recent research on imagery, mental modeling, mental simulation, and analogy, analyzing how those processes can work together as a system for creative heuristic reasoning to produce conceptual change. It also goes vertically upward, describing how Maxwell’s development of perhaps the most groundbreaking, abstract, and mathematical theory of the 19th century in physics was based on mental simulations and transformations of qualitative, concrete, analog models from mechanics and fluid mechanics, a remarkable story indeed. Thus her book extends the possible domain of the findings on model construction processes by a surprisingly large degree into a much more abstract domain.

The third book is a study of experts working on prediction and explanation problems in mechanics that complements the other two by using video-taped think aloud protocols to paint a more fine grained picture of scientific reasoning than is possible from historical studies (Clement, 2008). This allows more depth in the analysis of smaller model modifications, occasional Aha! episodes, and depictive gestures and other observations that were indicative of imagistic reasoning. Nersessian (2008) also made comparisons between Maxwell’s development process and one of the protocols in Clement (2008) and found a number of similar strategies. Each of the authors was motivated by the general long-term question of the strategies used for creative model construction in science. Because scientists encounter many interacting difficulties, it is a tangled and complex topic, where we have an inadequate qualitative theory of what the strategies are and how they interact, and where case studies can be an important source for developing an initial ‘field map’ of the area.

### Objectives

The first objective of this paper is to assemble a collection of reasoning strategies or processes that were used during scientific model construction in the three case studies. Due to space, I will necessarily leave out a number of strategies, but I will include important ones that played a role in the most innovative, successful discoveries.

A second objective is to ask whether the processes are organized some way, such as in a series or cycle. The organization question has had proponents on both sides. An anarchistic view with little structural organization for theory construction processes stems from Feyerabend’s (1975) book *Against Method*. Diametrically, in an influential book, Langley et al. (1987, 2006) believed they had demonstrated via AI programs that scientific discovery could be achieved using highly organized algorithmic methods. However they did not focus on the invention of new qualitative theoretical representations or the abduction of visualizable explanatory models, which will be foci here. The types and degree of organizational structure is a difficult question that will certainly not be settled in this paper, but I hope to develop a framework that can shed some light on the issue.

A third objective is to ask whether any of these expert scientific reasoning processes can be embodied or grounded in the perceptual and/or motor systems by examining descriptions in two of the books of the possible role of perceptual/motor imagery and mental simulations in modeling at the lowest level of the framework, also drawing on a think-aloud study by Trickett and Trafton (2007).

### Terminology

#### Grounded or Embodied

Unfortunately, the terms ‘embodied’ and ‘grounded’ have come to have many meanings (Wilson, 2002). While most of these meanings identify important topics, for purposes of focus in this paper I will use the term ‘grounded cognitive process’ in a narrow sense to mean that a cognitive process can utilize the perceptual and/or motor systems in the brain as a componential part of its operation, and will justify this later.

#### Heuristics, Strategies, and Processes

Polya (1954, 1957) and Lakatos (1976b) identified sets of strategies for mathematical problem solving and concept development. These were heuristics: ‘useful strategies to try,’ none of which are guaranteed to work, and I will retain that use of the term in this paper. *All of the reasoning methods described in this paper can be termed ‘heuristics’ or ‘strategies’ –or ‘processes’ when one wishes to emphasize their mental aspect– and I will use these terms largely interchangeably in this paper*. Ippoliti (2018), Cellucci (2020), Nickles (2019), and others (e.g., in Ippoliti, 2015) have recently revived philosophical studies of heuristics such as analogy, induction, generalizing and specializing and others, in science and especially mathematics. These provide encouraging directions, but there are still many open questions about what other heuristic reasoning strategies in science may exist, and how they might be organized. An interesting larger question concerns *how scientists can converge on a successful model by using heuristics that, individually, often don’t work*.

#### Models in Science

In this paper, I will use the term model to mean a mental representation that, given a target system, can be used to predict or explain the system’s structure or behavior by representing some initially unobservable parts, features, or relationships in the system. The usage here includes substantive analogies that are taken to provide possible insights on how a system works in terms of its hidden unobservable aspects. Early studies in history of science paved the way for a model based approach to understanding science, e.g., Campbell (1920/1957), Harre (1961), Hesse (1966), Giere (1988) and more recently on the related concept of mechanism (Machamer et al., 2000). These are complemented by studies of mental models in psychology (e.g., Gentner and Stevens, 1983; Forbus, 1984; Johnson-Laird, 2010) and important psychological studies of how individual processes like analogy can be used by experts to construct models in science, e.g., Gentner et al. (1997), Clement (1988), Catrambone and Holyoak (1989), Holyoak and Thagard (1995), Millman and Smith (1997), Casakin and Goldschmidt (1999), Dunbar (1999), Griffith et al. (2000), Nersessian and Chandrasekharan (2009), and Chan et al. (2012) (see also reviews by Dunbar and Fugelsang, 2005; Feist, 2006; Gorman, 2006; Thagard, 2012). But too little research exists on the relationships between creative scientific model construction and multiple types of heuristic reasoning.

#### Imagery in Science

A largely understudied aspect of creative scientific reasoning concerns the role of perceptual and motor imagery and mental simulation. Finke (1990) has shown how lay subjects can combine images in novel ways to produce new images with new interpretations. He defined imagery as “the mental invention or recreation of an experience that in at least some respects resembles the experience of actually perceiving an object or an event (Finke, 1989).” Here one can add the idea that this can include imagery of bodily forces or motions. When I say a subject is using *‘imagery’ here, I will mean either motor or perceptual imagery or both*, where motor imagery can include recreation or invention of kinesthetic perceptions, vicarious actions, and their anticipated results.

Miller (1984), Nersessian (1984), and Tweney (1996) were some of the first to argue that mental imagery use was important for scientists such as Faraday, Maxwell, and Einstein. Although important initial progress has been made on its role in specific problem solving tasks, e.g., Schwartz and Black (1996a), Schwartz and Black (1996b), Hegarty et al. (2003), Lowe (2004), Stieff (2011), and Reed (2019), there is still a dearth of information about how imagistic processes can support scientific modeling.

### Some Major Parallels in the Three Books

I need to begin by identifying some large scale parallels between the three book authors. First, each agrees that mental strategies used by scientists are embedded or situated in larger material, social, cultural, and environmental systems that play an extremely important role in what Gruber (1974) called their ‘network of enterprise.’ But in the three works of interest here, and much of their history, these authors have chosen to concentrate on a variety of poorly understood *cognitive* strategies, as a way of focusing their energy on a central part of the problem. I will have the same focus in this paper.

**FIGURE 1:**
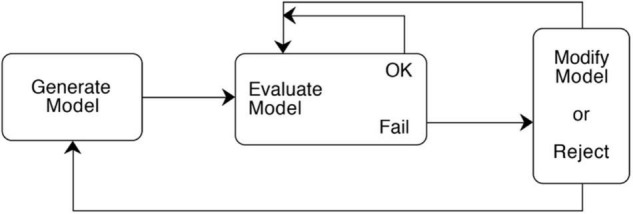
Model construction cycle of Model Generation, Evaluation, and Modification (GEM cycle). (1) A hypothesized model is generated. (2) The model is evaluated with respect to whether it plausibly explains target observations and whether there are any problems with the model in meeting certain scientific criteria, or conflicting with observations, constraints, or other theories. If it passes, other criteria may be found for further evaluation. If it doesn’t pass and the failure is fatal, the model is rejected, and one returns anew to the Generation process. Otherwise, a new constraint on the model can be noted and attempts can be made to modify the model within existing constraints. More than one modification may be made. (3) The modified model is evaluated, and the cycle of modifications and evaluations can continue until either the investigator gives up, or the model withstands evaluation sufficiently enough to satisfy the modeler for their purposes. (Reproduced with permission from Clement, 2008, p. 84).

Secondly, each of the authors has an approximately 40-year history of work on strategies used in science, and there is a central common finding that each of them converged on long ago. Each author describes the evolution over time of a scientific model that goes through a series of evaluations and revisions. I will describe this as a Model Construction Cycle– a pattern of Model Generation, Evaluation, and Modification (abbreviated: GEM cycle). The form of the cycle is described in the caption for Figure 1, basically indicating that a model can be constructed iteratively by an initial generation process, followed by a sequence of evaluations and modifications under constraints, unless there is a fatal flaw or major breakthrough that dictates starting over. Although this ‘GEM’ cycle is simply described, its power and centrality is supported by the fact that all three of the authors converged on its basic iterative form as central to model construction, despite working from quite different sources and using slightly different names for the three phases. [Darden (1991) describes this cycle as “(1) searching for new ideas, (2) assessing them, and (3) improving them” (p. 21), and Nersessian (2008) as a cycle of (1) Model construction, (2) Evaluation, and (3) Adaptation (p. 184). This illustrates one kind of consolidation in settling on compatible terminology that I needed to do for the consolidation.] Model construction cycles in science have also been discussed elsewhere, e.g., Shapere (1980), Nersessian (1984, 1992), Holland et al. (1986), Darden and Rada (1988), Goel and Chandrasekaran (1989), Forbus and Falkenhainer (1990), Gentner et al. (1997), Millman and Smith (1997), and Gorman (2006); and by Miyake (1986) and Clement (1989) for protocols.

Thirdly, I would characterize the three authors as analyzing detailed historical examples or protocol observations as significant constraints, to hypothesize reasoning patterns that could have produced those examples or observations. Qualitative case studies in psychology are not intended to report on data from large numbers of subjects, but the approach has considerable value in the early stages of a field area, especially in an area with highly complex or nested, interacting processes where qualitative theoretical mechanisms are sparse or uncertain (Newell and Simon, 1972; Cronbach, 1975; Campbell, 1979; Strauss and Corbin, 1998; Dunbar, 1999; Weisberg, 2006; Jaccard and Jacoby, 2010; Tweney, 2012; Ylikoski, 2019), including for in-depth psychological microanalysis studies with single subjects (e.g., Gruber, 1974; Anzai and Simon, 1979; Tweney, 1992; Gentner et al., 1997). The first desired outcome for this paper in the ‘hypothesis and theory’ category of this journal is to construct a qualitative theoretical framework or ‘field map’ of important scientific reasoning strategies for model construction, and major interconnections between them, on the basis of detailed case studies of scientific events.

### Four Levels of Processes and Plan for the Paper

To anticipate, as an advanced organizer, after reviewing strategies identified in the three case studies, a modeling processes framework describing a partial organization of the modeling strategies will be proposed at four nested levels:

• Level L4. An overarching set of five longer-time-scale *Major Modeling Modes*, for example Model Evolution, in which a model is generated and then improved iteratively, or Model Competition, in which two or more models compete.
• Level L3. Substrategies (Subprocesses) for implementing the central Model Evolution Mode above, namely *Modeling Cycle Phases* of Model Generation, Evaluation, and Modification (Figure 1), after Conducting Exploratory Observations. By substrategy, I mean a smaller strategy that is part of a larger strategy.
• Level L2. A set of 13 *Tactical Heuristic Strategies* that in turn act as substrategies for implementing strategies at level L3, e.g., Analogy, Running a Model to Evaluate It, Analyzing Extreme Cases, etc.
• Level L1. *Imagistic Strategies*, namely Mental Simulations of and Structural Transformations of a model, that can occur as substrategies within most of the tactical heuristic strategies at Level *L2.*

The plan for the paper is to describe: (1) strategies from the three case studies at levels L3 and L2 first since level L3 is central; (2) the proposed larger framework organization; (3) strategies assigned to levels L4 and L1; (4) grounding at the lowest, and then possibly higher levels; (5) possible benefits of this organization and grounding; (6) possible directions for future versions of the framework.

## Heuristic Scientific Reasoning Strategies Identified in the Case Studies

### Historical Case Study of Strategies Used in Genetics

A first set of strategies at Level 2 that implement the central Model Construction Cycle strategies at Level 3 can be obtained from Darden’s (1991) case study of the early history of genetics. It covers an approximately 30-year period of theory construction, from the rediscovery of Mendel’s work in 1900 to the 1930 version that is used in textbooks today. I can only describe a simplified portion of the heuristic strategies she identified here. Darden described her book as hypothesizing strategies that could have produced the historical changes she observed in the historical record. Table 1 illustrates three GEM cycles in column 3. Darden categorized each Level 2 strategy in column 2 (e.g., analogy) as one of the three GEM types in column 3 (e.g., ‘Model Generation’).

**TABLE 1:** Examples of strategies used in the development of genetics.

*Episodes: Darden (1991) on Genetics*	*Tactical Heuristics (L2)*	*Modeling Cycle Phases (L3)*
1. Mendel develops the inheritance of unitary ‘characters’ concept.	(Unknown; insufficient records)	**Model Generation**
- de Vries’ makes explicit Mendel’s simplifying assumption of the one to one relation between a visible character and the inferred underlying unit	**Simplifying Assumption**	

2. Researchers ask: By what mechanism are characters transmitted?	**Identifying a Gap in the Model**	**Model Evaluation**

3. Genes (‘factors’) are involved in the transmission of characters	**Addition of a Model Element** (‘factors’ or genes)	**Model Modification**

——————————	—————	** —————**

4. Mendel’s observed inheritance patterns and assortment theory of 9–3–3–1 ratios		Original Model

5. Discovery of linked traits with anomalous ratios	**Model Prediction Failures**	**Model Evaluation**

6. Bateson’s differential *reduplication* of germ cells *hypothesis* explains some linked trait cases	**Addition of Model Elements** (to Mendel’s theory)	**Model Modification**

7. But Morgan has criticisms of the above:		**Model Evaluations**
- Reduplication never observed	**Model Prediction** Failure	
- Presents additional anomalous ratio data	Other **Explanation Failures**	
- Required modifications to Bateson’s theory would be *ad hoc*, very complex, and could not extend easily to other cases	Discredited by **Refined Criteria for Theories:** Ad hocness, simplicity, extendibility	

*Competing response to Item 5 above*:		

8. Morgan utilizes exploratory observations made in cytology to hypothesize linked genes on chromosomes, like ‘beads on a string’ and crossing over, connecting the fields of cytology and genetics	**Using Interrelations with Another Field** **Analogy**	**Exploratory Observations;** **Model Generation**

9. Above model explains partial coupling of characters	**Features Inferred from Model Explain Relevant Observations**	**Model Evaluation**
- Predicts observing chromosomes breaking and rejoining during crossing over (Confirmed later)	**Successful Prediction and Testing via Evaluatory Observations**	**Model Confirmation**

*Heuristic reasoning processes are shown in bold.*

In what follows below, the more general model construction phases of the GEM cycle are capitalized in bold letters, while more specific heuristic strategies used appear in bold lower case (for those unfamiliar with the biology, the gist of the GEM cycle in column 3 of Table 1 and supporting tactical heuristics in column 2 are the most important findings here).

As shown in Table 1, **Model Generation** was initiated by Mendel in his research involving unitary, separable characters of pea plants, such as color and height. In expounding and clarifying Mendel’s theory, de Vries’ made explicit the **simplifying assumption** of the one to one relation between a visible character and the inferred underlying unit.

Mendel’s concept of a ‘character’ of the organism was eventually recognized as deficient because it did not include a mechanism explaining how characters are transmitted. This **Evaluation** of the model is described by Darden as **identifying a gap in the model**. It motivated researchers to propose the idea of a *‘factor’* (an early nascent version of the concept of the *gene*) that could be segregated, independently assorted, and associated with a germ cell that was transmitted to the daughter organism (I will use the more modern term ‘gene’ from this point on). The **Modification** process in this case was the **addition of a model element**. So far this provides a simple example of one round of the G-E-M cycle.

Mendel had also observed and explained 9–3–3–1 inheritance ratios for two traits of offspring from dihybrid crosses with independent assortment in peas. In the next cycle shown in Table 1, Bateson and others **Evaluated** the theory by finding cases with linked traits and anomalous ratios that deviated from the expected 9–3–3–1, exposing **failures in predicting new observations**. In response, Bateson and Punnett **Modified** the theory by **adding model elements:** their Reduplication Hypothesis specified differential duplication of some of the germ cells, producing unequal numbers of types of gametes, accompanied by elaborate diagrams of duplication patterns. As shown in Table 1, The Reduplication Hypothesis did explain the initial anomalies, but was eventually **Evaluated** as unsuccessful, in part because the **prediction** of differential cell division at early stages was never observed. In addition Morgan discovered coupled characters with other anomalous ratios exposing **failures to explain relevant observations**. He also argued that the necessary modifications to Bateson and Punnett’s reduplication theory to account for them would be so convoluted as to violate **refined criteria for theories** such as simplicity, extendibility, and avoidance of ad hocness.

In item 8 Morgan **Generated** his own, remarkably innovative, competing theory in response to the **prediction failures** in item 5 in Table 1. Utilizing the **Exploratory Observations** (observations not motivated by theory evaluation) from research on chromosomes in cytology, he hypothesized that linked genes were on the same chromosome, like the **analogy** of beads on a string, and that *strings* of linked genes on the same chromosome assort during meiosis rather than individual genes. Darden cites this as an example of also **using interrelations with another field,** here the previous observations of pairs of chromosomes in cytology. In addition he hypothesized that *crossing over* could occur with chromosomes able to exchange a portion of their chain of genes to explain partial coupling. This theory was **Evaluated** positively as having more explanatory adequacy than Bateson’s for **explaining relevant observations** of anomalous ratios of offspring types. Morgan also **predicted observing** that chromosomes break and rejoin at crossing-over points; cytologists eventually confirmed this visually in the 1930s.

In summary, we see an interesting variety of tactical heuristics used in column 2, Table 1. The overall pattern in column 3 of Table 1 shows a Modeling Cycle: a cyclical repetition of model Generations, Evaluations and Modifications which correspond to Figure 1. Darden describes such GEM cycles as central, and lists each of the smaller tactical heuristics within one of those three categories in her book summary^1^.

#### Consolidation Process for the Book Summaries

I selected examples for inclusion above as follows. In the left hand column of Table 1 there are numbered episodes, each of which contains one or more events. Each event is an example of a heuristic strategy in column 2. Events were selected from Darden’s book (and the others) for inclusion in the tables based on several criteria. The first problem was to find sequences of events that exemplified a variety of strategies, but where it was also possible to describe here in a short space, without too much technical background. The other criterion was to include some of the most impressive examples of innovative modeling and conceptual change (as a major change in the structure of a model or concept), made easier by the books being focused already on exemplary thinking. Thus this paper focuses on exemplary scientific modeling.

A second problem was to consolidate similar strategies identified by the different authors. For some items like ‘Simplifying Assumption,’ there was significant agreement by authors on both the concept and the vocabulary term. However for others, there was a need to consolidate concepts or vocabulary. To reduce complexity, closely related strategies where authors made only fine distinctions were merged together. Where different vocabulary was used by different authors for basically the same strategy, a consolidated term was sought that reflected their common meaning, and the consolidated terms are used here and in tables one through four.

### Think Aloud Case Study in Mechanics

**FIGURE 2:**
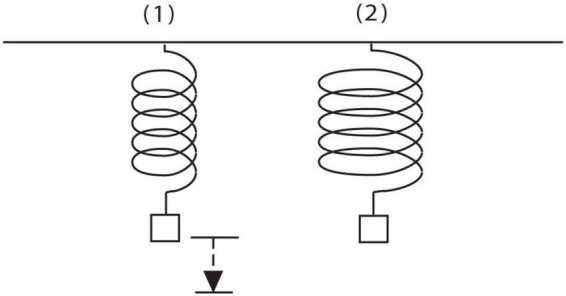
Spring problem. A weight is hung on a spring. The original spring is replaced with a spring made of the same kind of wire, with the same number of coils, but with coils that are twice as wide in diameter. Will the spring stretch from its natural length more, less, or the same amount under the same weight? (Assume the mass of the spring is negligible). Why do you think so? (Reprinted with permission from Clement, 2008, p. 26).

In Clement (2008) experts were videotaped working on unfamiliar explanation problems using think-aloud procedures (Ericsson and Simon, 1984). They were professors or doctoral students who had passed comprehensives in a variety of technical fields. One task used was the target problem in Figure 2. The reader may enjoy generating ideas on it, including informal ones, before reading further. I will focus here on the solution of a single subject S2, who produced the most productive solution and explanation for the spring problem, because it eventually will enable me to illustrate all four levels of the framework compactly. In the protocol, S2 not only predicts correctly that the wide spring stretches more, but also confirms that and answers ‘Why?’ by inventing a model for how a spring wire deforms during stretching and how it provides a restoring force back in return. He initially thinks that the spring wire is *bending*, but then detects a serious problem with that model. He eventually experiences an Aha! event and improves the model by hypothesizing that the wire is *twisting* when it is stretched. That discovery represents a conceptual change in identifying a new hidden variable and causal mechanism for stretching. It is also a marked conceptual breakthrough because the subject was stuck in the bending model for a considerable period of time, suggesting that he had to overcome an idea fixation or set effect. Twisting of the wire and the resulting torsional strain is in fact the most important source of stretching and restoring force recognized by engineers (Appendix I in Supplementary Material contains an introduction to the concept of torsion).

**FIGURE 3:**
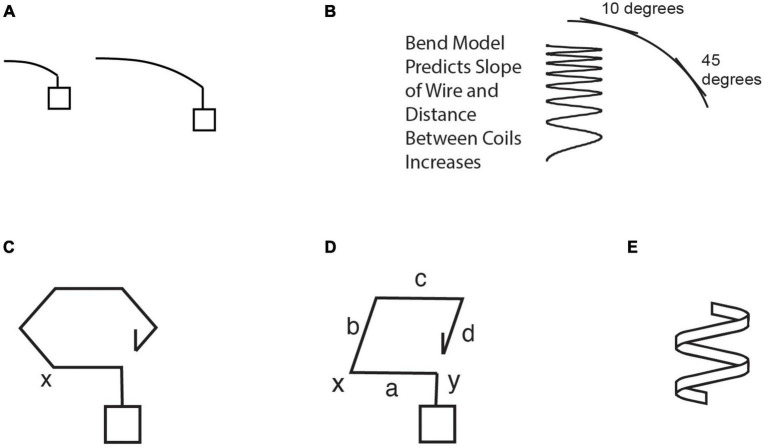
Sequence of models considered by subject S2 that led to a conceptual breakthrough in Clement (2008). Bold capitalized type below signifies major **Modeling Cycle Phases**; bold lower case signifies **tactical heuristic reasoning processes**. **(A)** S2 generated an **analogy** to long and short bending rods, and his mental simulation of the longer rod bending more implied that perhaps the wider spring would stretch more. (Panels **A,C–E** are redrawn from diagrams made by the subject.) **(B)** But using this analogy to **Generate a Model** of elements of the spring wire bending as it stretches leads him to **Evaluate** the model via a **conflict with relevant observations:** the slope of a bending rod increases along its length, and he infers this means the coils at the bottom would be farther apart, in panel **(B)**; whereas he knows that real springs stretch uniformly with a constant slope in their coils and equal distance between coils, which become heeded constraints. **(C)** After a long period of struggling with this conflict, he **simplifies** to a single coil and thinks of **Modifying** the model by **altering** the rod into a hexagonal coil model. He then has a sudden AHA in **inferring a new feature by mental simulation:** that the forces in it will introduce a *twisting* effect in the wire, not just bending. He also **applies a schema to the model** (here the scientific concept of torsion). **(D) Evaluating** the hexagon as **not simple enough**, he **Modifies** the model by **simplifying** it into a square coil model. (He appears to imagine the situation in panel **(D)** as if side ‘a’ were a wrench acting at ‘x’ to twist the end of side ‘b’ through an angle, while side ‘c’ keeps the other end of ‘b’ from turning in the same direction, resulting in a twisting deformation of the metal in side ‘b’). Via mental simulation he then sees that bending and twisting ‘start over’ at each corner and do not accumulate in a square spring. This allows **Evaluation** of the model by **explaining the relevant observation** of the equal space between coils constraint, resolving the earlier anomaly for this case. He then **Modifies** the model further by **inferring a new feature** by mental simulation– that a wider square spring will stretch more, explained by its longer sides experiencing both more bending and more twisting, confirmed by an **extreme case**. **(E)** Later he negatively **Evaluates** the bending part of the model with a **Gedanken (thought) experiment** via mentally simulating a spring made of a band of metal that “can’t bend…but can easily twist” as it stretches, indicating bending is not necessary for stretching. Here, conducting a Gedanken experiment means attempting to mentally predict the behavior of an unfamiliar, concrete system (the “experiment”) designed to help evaluate a scientific model (Clement, 2009b). (This Gedanken is from a second interview simulating empirical input where S2 was told that measurements show that the primary deformation in the spring segments is a twisting or torsion effect as opposed to bending, and asked to provide a further explanation or argument for that.) See also Table 2 for condensed transcript of this entire sequence.

The sequence of models generated by S2 is shown in Figure 3, and the caption describes how they emerge from phases of the GEM cycle and the smaller strategies used for each phase in it. A corresponding, condensed transcript is shown in the first column of Table 2. As the reader reads down the transcript there, items in bold in the Level L2 column show the tactical heuristics used to implement the model construction cycle in the Level L3 column (similar to the cycle in Table 1 for genetics). Levels L1 and L4 will be discussed later.

**TABLE 2:** Processes used for developing a model in mechanics. In column 5 G, E, M, = Model Generation, Evaluation, or Modification. In column 1 parentheses indicate subject’s actions; brackets indicate my clarifications. Underscores identify evidence for imagery use (kinesthetic or visual), described in column 2. Terms in bold in column 4 are Level L2 reasoning processes. Also see Figure 3 caption.

*Condensed Transcript Episodes*	*Specific Imagery Indicators*	*Level L1: Imagistic Processes*	*Level L2: Tactical Heuristic Processes*	*Level L3*
***1. Generating Bending Rod Analogy*** “A spring is nothing but a rod wound up…Maybe I could answer the question for a rod.		-Structural Transformation (Inverse description)	-Generates **Analogy** (which becomes a model when projected into the spring)	**G**

***2.*** But…there’s something wrong with that metaphor…If I took spring wire and it was straight [horizontal] instead…[with the weight] It would droop (moves r. hand to the right in a downward curve) like that and its slope would steadily increase as you…went away from the point of attachment; whereas in a [stretched] spring, the slope…is constant. (see Figure 3B and caption)	-Depictive gesture	-Imagistic Simulation -Simulation Outcome Comparison	-**Takes Feature** (increasing slope) **Inferred from Model** (by running Bending Model) and **Compares to Relevant Observations** (of springs, generating conflict)	**E**
***3.*** I’m imagining a…rod—(draws straight rod) when you hang a weight on it, my physical intuition says that what happens is it droops something like that. (draws downward curving rod). [But] if I had a real spring…it would just stretch uniformly…the distances between the coils would be equal… *[Long section here of reviewing variations of this same conflict until the following breakthrough*]	-Imagery report	-Imagistic Simulation -Simulation Outcome Comparison	-Repeats above evaluation for distance between coils	

***4. Hexagonal Coil Formation*** I can reduce…to a one-coil case maybe… What if I start with a rod and bend it once (motions as if bending a wire) and then I bend it again…What if I produce…polygons! Maybe that would clarify…(draws hexagonal coil Figure 3C)	-Depictive Gesture	-Structural Transformation -Imagistic Structural Transformation	**-Simplifying Assumption** -**Alters Model** to Hexagonal Coil	**M**

***5. Twisting/Torsion* *Insight*** Interesting, just looking at this [hexagon drawing] it occurs to me that when force is applied here, you not only get a bend on this segment, but because there’s a pivot here (points to x in Figure 3C), you get a *torsion* effect… **Aha!!…** The spring has something to do with *twist* (moves hands as if twisting an object) forces as well as *bend* forces (moves hands as if bending an object)…	-Describes invisible feature in drawing -Describes invisible feature in drawing -Depictive Gesture -Depictive Gesture	-Imagistic Simulation -Imagistic Simulation -Imagistic Simulation -Imagistic Simulation	**-Infers New Feature of Model** (by Running It) -**Applies Schema** (Torsion/Twisting)	**M**

***6. Square Coil*** Let me accentuate the torsion force by making a square…(Draws a square, Figure 3D) that unmixes the bend from the torsion…	-Describes invisible features in drawing	- Structural Transformation	-Hexagon not **Simple** enough (**Refined Criteria**) -**Simplifies Model** to Square Coil	**E** **M**

***7. Inferring New Features of Model*** (a) Does this gain in slope–toward the bottom?…Each individual segment gains in slope (moves hand horizontally in a downward curve)…[but it] starts over again at each joint…we have a structure here which does not have this increasing slope…(moves hand in downward curve)…The curving…is local within each segment. (b) Now making the sides longer certainly would make the [square] spring stretch more…the longer the segment (moves hands apart) the more the bendability (moves hands as if bending an object)… (c) [Also] If I have a longer rod [side] and I put a twist on it (moves hands as if twisting something), it seems to me–again physical intuition–that it will twist more	-Depictive Gesture -Depictive Gesture -Describes invisible events in drawing -Depictive Gesture -Depictive Gesture -Describes action projection -Depictive Gesture	-Imagistic Simulations -Imagistic Simulations and Comparison -Imagistic Simulations and Comparison	**-Evaluates Model (by Running It**)-to **Explain Relevant Observations** (Resolves earlier anomaly: square spring doesn’t accumulate slope) **-Infers New Feature of Model** (by Running Component) -**Infers New Feature of Model** (by Running Component)	**E** **M**

***8. Twisting an Extremely Short Rod*** Now I’m confirming that…(moves right hand slowly toward left hand until they almost touch). As I bring my hand up closer, I realize very clearly that it will get harder and harder to twist. So that confirms my intuition so I’m quite confident of that…	-Depictive Gesture -Kinesthetic imagery report	-Imagistic Simulations and Comparison	-**Extreme Case**; (to evaluate previous simulation)	**E**

**9. Band Spring Gedanken Experiment** We’re trying to imagine [spring-like] configurations that wouldn’t bend. (Draws Figure 3E) Since it’s cross section is like that…it can’t bend in the up-down (indicates up/down directions with hands) direction like that because it’s too tall. But it can easily twist (motions as if twisting an object).	- Reports Seeking Imagery -Depictive Gesture -Depictive Gesture	-Seeking Imagistic Structural Transform within Constraints -Imagistic Simulation -Imagistic Simulation	**-** Invents **Gedanken Experiment** (a vertical band spring, to evaluate bending model)	**E**

**FIGURE 4:**
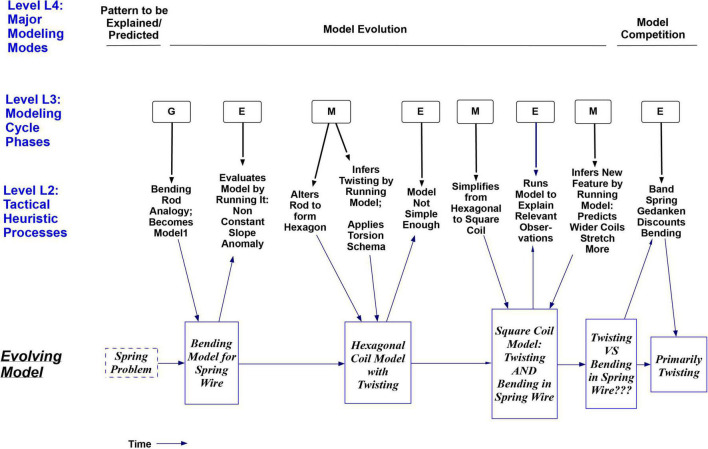
Time sequence diagram for three levels of model construction processes used by S2, and the resulting sequence of models. The row labeled Level 3 uses the letters G, E and M to show the Generation, Evaluation, or Modification phase of the GEM Cycle described earlier in Figure 1. The cycle is seen here as generating an alternating pattern of Evaluations and Modifications. The row labeled Level 2 shows the Tactical Heuristic Processes that helped to evolve the model in each of the above phases. These are seen as subprocesses for each phase at Level 3, as indicated by the thick black downward arrows, which mean ‘Utilizes the Subprocess’. The bottom row in the figure does not show reasoning processes or a process level, but rather the progression of new models resulting from the processes above it, as indicated by the thin blue downward arrows from Level 2 to the models underneath. The top row shows major Model Construction Modes to be discussed later, including a Model Competition Mode for deciding whether bending or twisting is the dominant model for deformation in the spring.

Figure 4 shows a summary of three of the levels of reasoning processes (strategies) used by S2. The rows labeled Level L3 and Level L2 show several Model Construction Cycles as the initial bending model was Generated, then thrice Evaluated and Modified, until it included twisting as well. The thick downward arrows in Figure 4 also show the sense in which the more specific Tactical Heuristic Reasoning Processes at Level 2 can be considered *subprocesses* for the more general Modeling Cycle Phases at Level 3; each subprocess at Level L2 contributes to the larger process above it. Later the subject distinguishes between confidence in his *answer* to the spring problem, which has been high, and confidence in his *understanding* of it, and estimates that his torsion idea has increased his understanding of the system from “way, way down” up to “like, 80%.”

In conclusion we see that for levels L2 and L3, S2 is using several of the same strategies identified in the genetics episodes in Table 1, including the three parts of the GEM cycle, plus Analogy, Simplification, Addition of a model element, and evaluation of whether it Explains relevant observations. These and other Tactical Heuristic Reasoning processes enabled him to make a conceptual breakthrough in an Aha event and to reconceptualize his explanatory model for the spring system^2^.

### Historical Case Study of Maxwell’s Construction of the Theory of Electromagnetism

A similar model construction cycle was described by Nersessian in the case of Maxwell. I will not go into his mathematical results, but outline the series of qualitative mechanical models he used since that is the focus here and for Nersessian (2008). Nersessian’s book weaves together historical analysis and a survey of previous psychological research on mental modeling, mental simulation, analogy, and thought experiments, analyzing how those processes work together for creative reasoning and conceptual change.

**FIGURE 5:**
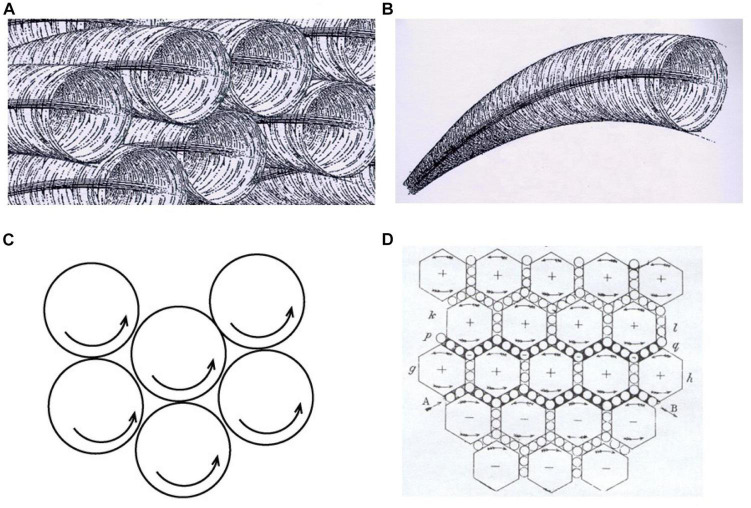
Maxwell’s development of E&M theory via analog models. Below, specific tactical heuristics appear in lower case bold and model construction phases of the GEM cycle appear in capitalized bold letters. Starting from Faraday’s ‘lines of magnetic force’ around a magnet, the (simplified) reasoning processes for Maxwell are: **(1) Model Generation** (via **using an analogy**): He imagined that the magnetic field is like a fluid with vortices **(B)** rotating in the same direction around each of the magnetic lines of force (**A** - Nersessian’s rendition). This qualitative model, along with incorporating a continuum mechanics theory of fluid flow (**applying a schema**) inspired an initial mathematical model for basic magnetic phenomena, as summarized in Table 3. **(2) Model Evaluation** (by **evaluating internal incoherence via running the model**): Mentally simulating the above system, Maxwell believed adjacent vortices would die out because of friction generated between vortices, just as gears turning in the same direction will jam if they touch **(C)**. This cast doubt on the initial model and became a constraint. **(3) Model Modification** (by **analogy** and **adding a model element**): Maxwell transformed his model by adding small vortices between the larger vortices, analogous to ‘Idle wheels’ between gears, to enable the vortices to rotate without jamming or creating friction **(D)**. He makes the **simplifying assumption** that fluid vortices are inelastic and do not deform. This provided a way to model electromagnetic induction (the principle of the generator) and electric current in a wire. **(4) Model Evaluation** (by **identifying a gap in the model**): Maxwell found a gap in the above model in not being able to account for static electricity and other phenomena when running the model in a mental simulation. **(5) Model Modification** (by **altering a model element**): He transformed the model again by adding elasticity to the vortices, yielding a way to model the above phenomena. **(6) Model Evaluation** (by **running the model to predict new Evaluatory Observations**): Mental simulation allowed him to predict the propagation of electromagnetic waves through space (also from equations). Confirmation of this prediction after Maxwell’s death by Hertz’s discovery of radio waves was a sensational contribution to confirming Maxwell’s theory (see summary in Table 3). (From Nersessian, 2008 p. 138, © 2008 Massachusetts Institute of Technology, by permission of The MIT Press).

The Figure 5 caption describes a progressively more complex series of qualitative analogical models constructed by Maxwell on his way to developing his final equations. The sequence in Figure 5 represents three passes through a cycle of model evaluation and modification, as summarized in Table 3, right-most column. Notably, Nersessian shows how tracking and accumulating constraints is important. Negative evaluations often provide constraints for the next modification process, not just disconfirmation. This is especially important for Nersessian in dealing with Maxwell’s case, because the mechanical models suggest abstract constraints that he can reflect in his mathematical models, via a Generic Abstraction process, even though he must in the end give up most or all concrete aspects of the mechanical models he used. Another strategy is *Apply Schema(s) to a Model*. This refers to accessing and applying one or more schemas that have not been applied to the phenomenon before. Maxwell adapting ideas from the new science of continuum mechanics to E&M theory is one example. Nersessian also finds many parallels between processes she identifies in one of the Clement (2008) protocols and Maxwell’s processes; this is also seen in Tables 2,3^3^. In sum, even though Maxwell’s objective was to reach a quantitative theory at an extremely high level of abstraction, Nersessian argues that his development of the theory, remarkably, depended on a series of visualizable qualitative models generated by a GEM cycle.

**TABLE 3:** Examples of processes used in development of Maxwell’s theory of electromagnetism.

*Episodes: Nersessian (2008) on Maxwell*	*Tactical Heuristic Processes (L2)*	*Modeling Cycle Phase (L3)*
1. Proposes analog model, transforming Faraday’s magnetic field lines into fluid-like Vortices to account for basic magnetic phenomena	**Analogy**	**Model Generation**
- Incorporates continuum mechanics of fluid flow	**Applies Schema to Model**	

2. Has problem of Vortices stopping from friction (like Gears Jamming)	**Evaluates Internal Coherence** by Running Model	**Model Evaluation**

3. Adds additional Vortices acting like ‘Idle Wheel Particles’ and formulates equations for electromagnetic induction and current	**Analogy** **Addition of Model Elements**	**Model Modification**
- Assumes fluid Vortices are not elastic. (i.e., do not deform in interactions)	**Simplifying Assumption/Idealization** of model elements	

4. Problem: Unable to formulate equations for static electricity	**Identifying a Gap in the Model**	**Model Evaluation**

5. Adds elasticity to Vortices and formulates corresponding equations for static electricity and other phenomena	**Altering a Model Element**	**Model Modification**

6. Runs model to predict possibility of electromagnetic waves (also from equations), later confirmed by Hertz	**Prediction** (from Running Model) (also from equations) and **Testing via Evaluatory Observations**	**Model Evaluation**

		

*Heuristic reasoning processes are shown in bold.*

## Theoretical Framework for Four Levels of Modeling Processes

### Examination of Levels 2 and 3 of the Framework

Thus far a consolidated set of important modeling strategies from the three authors has been examined at Levels L3 and L2 in Tables 1–3, as follows:

• *For Model Generation*: Simplify or Idealize; Analogy; Apply Schema(s) to Model; Interrelations with Another Field.
• *Model Evaluation*: Evaluate Model on Constraints & Internal Coherence (e.g., by Running it); Take Features Inferred from Model (e.g., by Running it) and Ask if it: (A) Explains Observations, or (B) Predicts New Evaluatory Observations; Identify Gap in Model; Extreme Case; Gedanken (Thought) Experiment; Evaluate on Refined Criteria, e.g., Simplicity, Ad Hocness, Extendibility.
• *Model Modification*: Add, Subtract, or Alter Model Element within Constraints; Analogy; Infer New Feature of Model, e.g., by Running It; Apply Schema(s) to Model; Simplify Model.

**FIGURE 6:**
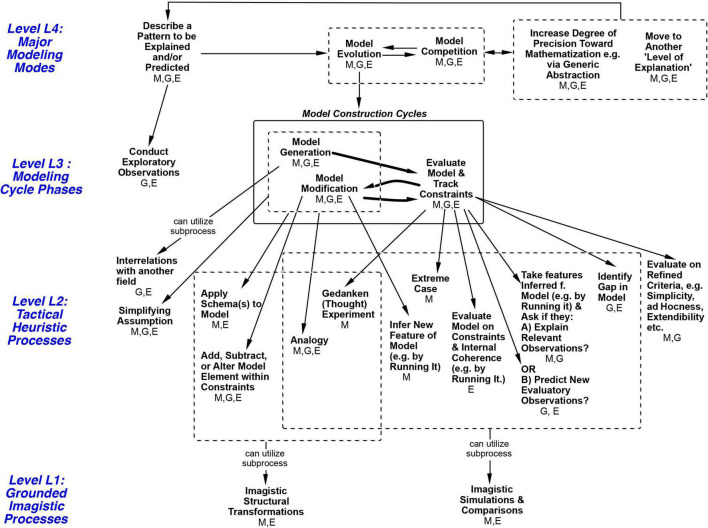
Modeling Processes Framework with four levels of reasoning processes. The framework represents a process hierarchy. Higher levels are hypothesized to contain larger tasks at longer time scales. Horizontal arrows show possible sequences. Arrows between levels mean ‘Can utilize subprocess’. A subprocess can contribute to a larger goal/process above it. For example, S2’s episode 2 in Table 2 is an episode of Model Evolution at Level 4, implemented in part by Evaluate Model at Level 3, which is in turn implemented by the subprocess Ask if Model Explains Relevant Observations at Level 2, implemented via a Mental Simulation comparison at Level 1. The GEM cycle at Level 3 can be envisioned as accessing and alternating between processes on the left and right sides below it at Level 2. At Level 2, within each set (such as those under ‘Evaluate Model’). the processes are not shown in any necessary order. All processes in the figure are heuristic in being not guaranteed to work, although ‘Tactical Heuristic Processes’ is used to name Level 2 because items at that level are historically most associated with the term ‘heuristics’. Initials under each process indicate fields where it was seen in this paper: **M, Mechanics (S2); E, Electromagnetism (Maxwell); G, Genetics**. The great majority of processes occurred in more than one field; this provides an initial indication that those are field-general processes (others may turn out to be as well). Note that at: **Level L4:** Only Model Evolution processes at Level 4 are unpacked in this diagram. **Level L3**: Evaluation, for example, can be implemented by any of the seven subprocesses it is pointing to, in any order, singly or with more than one being used. **Level L2**: L2 subprocesses for Model Evaluation at L3 are a different subset from the others at L2, indicating more structural guidance in the framework. Each Level 2 process is shown as a subprocess for only one process at Level 3, except for Analogy, Simplifying Assumption and Applying a Schema, which were found to occur with either Generation or Modification here (indicated by arrow originating from the dotted box at Level 3). **Level L1:** Similarly, the downward arrow from a dotted box at Level 2 to Level 1 indicates that the Level 2 processes within that box can each utilize the Level 1 strategy as a subprocess (to avoid drawing a profusion of arrows).

The importance of the strategies was indicated by their significant participation in episodes of insightful model construction and conceptual change. I can now propose the Modeling Processes Framework in Figure 6, which depicts an organizational structure for the above strategies at levels L3 and L2 and more, as described in the caption. The framework is constrained by the historic and protocol events analyzed in this paper from the three books, and it outlines a set of 24 processes that could produce them, drawing on the three authors’ hypotheses wherever possible. Again, all 24 are considered processes, strategies, and heuristics, so those terms are used more or less interchangeably here. The four levels shown were introduced in the “Abstract” and the “Introduction” sections.

As a starting point the GEM cycle shown at Level 3 in Figure 6 (abbreviated from Figure 1) represents a major convergence between the three authors. Additional structure can be added by hypothesizing that heuristics come in several different task ‘sizes’ and can be placed in corresponding levels. Craver (2007) and Wright and Bechtel (2007) note that the term ‘levels’ has been used in many ways. Here I use downward arrows to signify that larger processes *at a higher level of organization are implemented via smaller subprocesses at a lower level* (as in Figure 4). For example, the arrows from a process at Level L3 to Level L2 in Figure 6 indicate ‘subprocesses to try’ at Level L2, any one of which may or may not be sufficient, as ways to implement the process at level L3. Higher level processes are also hypothesized to typically take more time (Newell, 1990; cf. Nathan and Alibali, 2010). The figure also shows that the great majority of processes occurred in more than one field (see caption), providing an initial indication that those are field-general processes. Interestingly, the L2 strategies serving Evaluation at L3 are separate from those serving Generation and Modification. Thus, with many unordered subprocesses, the framework depicts a partially organized structure for heuristic modeling processes. It focuses on the processes identified in Tables 1–4 and certainly does not attempt to describe all processes used in science. *From here I will use the shorthand L1 to L4 to designate levels.* The framework builds on a smaller subprocess hierarchy framework for L1, L2, and L3 described in Clement (2008, p. 479). Two-level organizations at L2 and L3 were described in Nersessian (p. 184–5) and more broadly in Darden (1991, Chapter 15). All of the authors make the point that more than one L2 process can sometimes be involved in achieving a goal within a single L3 phase (as seen in Tables 1–3). Although none of the three books proposed a separate Level 4, each of the authors described most of the processes that I have shown there, to be discussed in the next section, followed by Imagistic Processes at Level 1.

**TABLE 4:** Four major modes of modeling that complement the Model Evolution mode focused on in this paper, as shown in Figure 6 at Level L4.

Major Modeling Mode at Level 4 in Figure 6	Genetics in Darden (1991)	Mechanics in Clement (2008)	Maxwell’s EM in Nersessian (2008)	Additional Comments
**Describe a Pattern to be Explained and/or Predicted:** The expert describes or is presented with a question that calls for an explanation or prediction by developing an explanatory model.	Mendel had identified a Pattern to be Explained in documenting interesting ratios of offspring in pea plants as a set of Exploratory Observations.	In the spring protocol, the pattern to be predicted and explained was defined by the prediction and explanation problem given to subjects.	Maxwell started from Faraday and Thompson’s Exploratory Observations as a pattern to be explained and also wanted to account for observations of all other electro-magnetic phenomena.	This process can focus on either an Observation pattern or a previous model that in turn needs to be explained in a deeper way by investigating how its components work.
**Model Competition**. This can occur as ‘inference to the best explanation’ when one or more experts realize that there is more than one model of a phenomenon and that only one model may be correct.	Morgan’s competitive theories vs. Bateson’s for explaining anomalous ratios. Thagard (1989) has studied local connectionist simulations for how networks of coherence and dissonance relations settle on a dominant model.	S2 considered whether the spring should be modeled solely as bending or twisting and created a Gedanken experiment to attempt to resolve this (Clement, 2008, 2009b) (See: Figure 3E caption; episode 9, Table 2; right-most item, Figure 4.).	After Maxwell published his theory, there developed an intense competition between a group theorizing an ether through which waves could travel, and others arguing against the ether, who were vindicated by Einstein’s special theory of relativity and its confirmation.	Possibly the Model Competition mode could be unpacked by hypothesizing that scientists primarily use the heuristics at L2 on the Evaluation (right-hand) side of Figure 6 to compare models while weighting each one, including the Refined Criteria there.
**Increase the Degree of Precision Toward Mathematization.** Model precision can be increased in stages toward becoming a mathematical model.	Accurate diagrams were a stepping stone aiding the calculation of offspring phenotype ratios.	Stages of precision leading toward mathematization: 1. pattern to be explained; 2. qualitative model elements; 3. imageable, causal mechanism aligned and connected spatiotemporally; 4. geometric model; 5. quantitative model. (S2 exhibits 1, 2, and partially 3).	Maxwell moved from concrete qualitative models to very abstract mathematics, via ‘Generic Abstraction.’ Remarkably, he was able to discern abstract constraints in the qualitative models and transfer only these abstract constraints to the equations.	Setting a goal for Increasing Model Precision, e.g., to a quantitative level, can also entail collecting data at a higher level of precision, hence the arrow feeding back from ‘Increase Degree of Precision’ to ‘Describe a [New] Pattern to be Explained’ in Figure 6.
**Move to Another ‘Level of Explanation’** to Initiate Modeling or Integrate with an Existing Model. (‘Level’ has a different meaning here).	Connecting genetic theory to chromosomes at a more microscopic level (also an example of using ‘Interrelations with another Field’ [cytology]) to help generate a model and predict crossing over.	A subject (not S2) posed a bending model, and then proceeded to explain the restoring force in a bending rod as tension in the upper half of the rod and compression in the lower half.	Maxwell taking Faraday’s electrical and magnetic field models as a Pattern to be Explained more deeply can be seen as Moving to Another ‘Level of Explanation’ from Faraday’s.	The above arrow also applies to Move to Another ‘Level of Explanation,’ where a model, once developed, can be taken as a pattern to be explained more deeply, requiring data at a different grain size.

Lindley Darden and Nancy Nersessian were kind enough to critique a draft of this paper, and approve of the summaries of important strategies from their work as sources contributing many items to the framework in Figure 6, while understanding that I necessarily could only represent a condensed portion of their analyses in the space allowed here (e.g., Figure 6 does not include the additional strategies listed for each author in footnotes to their summaries above). Any remaining errors are of course my responsibility.

### Level 4: Larger Model Construction Modes

Figure 6 shows several large-time-scale modes of scientific modeling hypothesized to operate at Level 4. So far I have considered only the Model Evolution Mode, which, as the main mode focused on in this paper, is the only mode that is unpacked in the framework at the lower three levels. For that reason the other four modes at Level 4 are only briefly described in Table 4 with examples. These modes are:

1. *Describe a Pattern to be Explained and/or Predicted:* Typically the expert identifies an observation pattern that is to be explained or predicted with an explanatory model.
2. *Model Competition:* When more than one model is constructed a choice can be made via comparisons on multiple criteria.
3. *Increase the Degree of Precision and Abstraction Toward Mathematization:* Once a rough qualitative model has been developed, its precision can be increased in stages toward becoming a mathematical model.
4. *Move to Another ‘Level of Explanation’:* (‘Level’ has a different meaning here). Given a constructed model (e.g., of tissue), one may decide to unpack and model some of its parts (cells) in more detail, or show how it explains functions at a higher level of explanation (an organ).

Once a qualitative model has support, a modeler can pursue any of the processes to the right of Model Evolution at L4 in Figure 6, until they reach a level of satisfaction that fits their purposes at hand. Adding the last two modes in the list above yields three possible (nested) cycles in the framework: the GEM cycle at L3, operating within the larger ‘Degree of Precision’ and ‘Level of Explanation’ cycles at L4 (Clement, 2008). This means the GEM cycle and processes below it are hypothesized to still be important as mathematical precision is increased, but with parameter manipulation and deduction (and recently computer simulation) becoming increasingly important at L1.

### Smaller Imagistic Processes at Level 1

The downward arrow from a dotted box at L2 to L1 in Figure 6 indicates that the L2 processes within that box can each utilize the L1 process below it as a subprocess. I first describe these L1 processes and then summarize arguments that they are imagistic processes.

#### Three Types of L1 Processes

Current researchers are still grappling with the constructs and terminology to describe mental simulations and related processes, and their use of terms is inconsistent. Both Clement’s (2008) and Nersessian’s (2008) books attempted to clarify, distinguishing between three different constructs that others have sometimes associated with the term ‘Simulation.’ I will use a slightly modified version of their terminology here.

**(Novel) Mental Simulations** of a System: In it’s most basic meaning, a subject using a Mental Simulation of a system makes a prediction or infers new results about a system changing from one state to another. This meaning spans a long literature (e.g., de Kleer and Brown, 1983; Forbus, 1984; Collins and Gentner, 1987; Clement, 1994). For example, a spring problem subject (not S2) in Clement (2008) said, “If we had a case [with] huge diameters compared to the first, it would appear to sag a lot more. It just feels like it would be a lot more spongy.” This is also a case where the subject *compares* two simulations, as is also seen in Table 2, episodes 7–8 for S2. In this paper I will be referring to *new* simulations that are personally novel for the subject rather than those generated by an established schema operating on a familiar question and domain of application for the subject. Note that this is a much narrower, more specialized construct than some others,’ such as Barsalou’s (2008, p. 618) “Simulation is the reenactment of perceptual, motor, and introspective states acquired during experience with the world, body, and mind.” For example the latter includes simply recalling what a rose looks and smells like, fitting his initial purpose of analyzing the nature of knowledge concepts, whereas the focus here is on innovative reasoning processes.
**Structural Transformations** of a System: A mental Structural Transformation occurs when the subject contemplates making a change to the structure or design of a system. Here these are typically used to assemble or modify a model or case, not to make predictions or inferences from it (e.g., if you were designing a ‘kinder gentler’ mousetrap, you might use Structural Transformations to imagine arranging, combining, and modifying parts for it, whereas you could then use a Mental Simulation to mentally predict its operation after being triggered by the mouse.)
**Basic Spatio-Physical Reasoning Skills:** These involve simpler perceptual transformations such as mental object rotations and the ability to reason internally about basic properties of objects and constraints on their movements from obstacles, etc., supporting everyday object manipulations and event perception. Lin et al. (2022) reviews their development from infants through adolescence. Kosslyn (1980) and Shepard and Cooper (1982) document adult subjects’ abilities to imagine rotating, translating, scaling, zooming, etc. of images. Whereas Structural Transformations change a structure, Spatio-Physical Reasoning usually conserves structure. Because Spatio-Physical Reasoning skills are very common, often automatic, can be used to support the above two processes, and are therefore hard to analyze separately, *I do not focus on them here or in Figure 6, but mention them to help distinguish them from the two more restrictive terms above.*

#### Level 1 Processes in Clement

Previous research has established widespread agreement that both perceptual and motor imagery exists (Kosslyn, 1994; Decety, 1996). There is also some evidence that it can be involved in some basic types of reasoning (Reed, 2022) and innovative combinatorial design (Finke et al., 1996). In Clement (2008) I used observable imagery indicators, described in Table 5, to provide evidence that subjects were using imagery, and provide increased support when more than one were observed together. Many examples of these imagery indicators appear in column 2 of Table 2 and refer to underlined observations in the S2 transcript. Most examples there refer to actions, forces, events, or changes, as indicators of dynamic, not just static, imagery. Most indicators coincide with mental simulations or transformations at L1 in column 3 of Table 2. This supports referring to these as cases of Imagistic Simulation and Imagistic Structural Transformation. A major function of imagistic simulations seen in the transcript is that an *imaged model can be ‘run’* in an Imagistic Simulation to *generate new inferences* from the model as a prediction or explanation, such as in episode 5, where S2 runs an Imagistic Simulation of the hexagonal coil. A major function of Imagistic Structural Transformations is to *modify a model*. The L1 imagistic processes in column 3 of Table 2 are seen as subprocesses that can often implement the Tactical Heuristic Processes in column 4 throughout the transcript.

**TABLE 5:** Imagery indicators in S2 protocol.

Imagery Indicator	Description	Example (from S2 Protocol in Table 2)	Comments
**Spontaneous imagery reports**	Describes; (1) imagining, seeing, sensing, or feeling something not present; (2) imagining vicarious actions; (3) efforts to imagine	Section 3: “I’m imagining a rod” Section 8. “As I bring my hand up closer, I realize very clearly that it will get harder and harder to twist”	To be spontaneous these must occur without the interviewer asking about imagery
**Depictive gestures**	Gesture that depicts a shape or event related to the problem at hand (excluding pointing to a diagram or stylistic gestures such as the “thumbs up” sign)	Section 4: Motions as if bending a wire	Depictive gesture appears to be a natural way of expressing mental imagery (McNeill, 1992; Hegarty et al., 2005; Clement, 2008; Hostetter and Alibali, 2019)
**Describes invisible element in diagram**	Describes a relevant concrete feature or event in a diagram, that is not pictured	Section 5: “When force is applied here, you…get a bend on this segment”	Diagrams may replace some imagery but do so without detail and cannot fully depict dynamic events
**Action projections**	Refers to actions of entities in a system as if they were conducted by a person	Section 7c:” If I have a longer rod [side of square coil] and I put a twist on it…”	These can indicate dynamic motor imagery

#### Level 1 Processes in Nersessian

Nersessian (2008) reviewed research on imagery and mental simulation and used her analysis of one of the Clement protocols to support her assertion that mental simulations and transformations are imagistic processes. She argues that Maxwell’s development of his extremely abstract equations relied on inferences from qualitative analog models of the motions of vortices and gears, and argues that these most likely took place via imagistic mental simulation because of his references to novel moving systems that he could not build and therefore must have somehow imagined. Another argument was that Maxwell had included in his papers diagrams of vortices with “text for how to animate it imaginatively, thus simulating a range of future states” (Nersessian, 2008, p. 134.) For other examples, *her analysis can be pictured as a Level L1 column inserted in between columns 1 and 2 in Table 3, where the cells in rows 1, 3, and 5 would contain Structural Transformations, and 2, 4, and 6 would contain Mental Simulations* (Nersessian, personal communication).

#### Level 1 Simulation Process in Trickett

In a series of studies, Trickett, Trafton, Schunn and others studied the use of “conceptual simulations” by experts thinking aloud while doing data analysis tasks in their own field, given data mapped out spatially on a computer display. In Trickett and Trafton (2007) eight pairs were studied from the fields of astronomy, fluid dynamics, laser pellet fusion dynamics, fMRI brain imaging, neural spikes research, and cognitive psychology, and 32 simulations were documented. For example, an astronomer imagined two moving groups of stars ‘bending’ in different directions in a mental simulation to explain observed, anomalous, interstellar gas flow maps. They write that conceptual simulations “occur in a mental representation that is an analog of physical space.” They describe that they involve three steps: “visualize a situation,” “carry out an operation on the visualization,” and “inspect the visualization (see what happens)” and that simulations are inferential in producing a new result. Thus they argued that simulations take place via visualizations and gathered evidence indicating that inferential simulations could be used by scientists in a variety of different fields. (They also found several other strategies associated with the scientists’ hypothesizing: tie-in with existing theory, design empirical test, analogy, and consult colleague, in addition to identifying data patterns).

### Can Any Expert Scientific Reasoning Processes Be Grounded?

In posing this question I noted that the terms ‘embodied’ or ‘grounded’ have come to have many potentially confusing meanings (Wilson, 2002). *Here I will focus on one meaning– that a cognitive process can utilize the perceptual and/or motor systems in the brain as a componential part of its operation*. I will use the term ‘perceptual-motor grounding’ or ‘grounded’ for short with this meaning to help avoid confusion here with other meanings of ‘embodied.’ My focus on it fits the sources I am analyzing, and it is not at all to deny the importance of others investigating other embodiment and ‘4E’ issues.

There is now widespread agreement that *visual* imagery is grounded in the sense that it uses the perceptual systems in the brain, based on considerable evidence as reviewed in Kosslyn (1994), Kosslyn et al. (2001), Svensson and Ziemke (2004), and Ganis and Schendan (2011). Schendan and Ganis (2012) conclude that “direct neural evidence reveals that top-down processes of mental imagery sustain an imagistic representation that mimics perception well enough to prime subsequent perception and cognition.” Reviews by, e.g., Jeannerod (1994, 2001), Decety (1996, 2002), Gre‘zes and Decety (2001), and Kilteni et al. (2018) provide similar conclusions for *motor* imagery as vicarious motor actions that can participate in mental simulations. Together these studies conclude that perceptual and motor imagery involve and make substantial use of largely the same perceptual and/or motor regions in the brain as used in real perception and action.

The above findings imply that when the Mental Simulation and Structural Transformation processes utilize imagery (perceptual and/or motor), they are grounded in the sense of utilizing the perceptual and/or motor systems in the brain. These two processes shown at L1 in Table 2 occur throughout S2’s protocol segments in conjunction with multiple types of imagery indicators. On the basis of this and other protocol data in Clement (2008), I concluded that these two L1 processes can utilize imagery, and we have substantial evidence from those cited above that imagery is grounded. Nersessian (2008) argued that Maxwell used these two imagistic processes, and Trickett and Trafton (2007) argued that scientists from six other fields were using dynamic, visualized mental simulations in data analysis tasks. Thus there are reasons for believing that the L1 reasoning processes of mental simulations and structural transformations can be grounded in the perceptual and/or motor systems of expert scientists. This conclusion, if true, is very much at odds with older traditional computational accounts of cognition, in that at least part of the knowledge representations are imagistic and modal, rather than consisting of amodal, language-like symbols, and that these are analog in preserving at least rough, schematic correspondence relationships to the spatial forms or behaviors of the entity being represented. In addition, reasoning can be conducted by dynamic processes such as mental simulation that coordinate imagery of actions or events that unfold over time, as opposed to reasoning via discrete logical operations or rules on static symbols. An implication is that the cognitive system does not always operate independently from the perceptual and motor systems, but rather can be closely intertwined.

One might ask whether the above argument is pre-empted by Barsalou’s all-encompassing theory that implies that *all* concepts may be grounded, modal ‘simulators.’ However, Barsalou states that his theory is controversial and in need of evaluation, especially with respect to advanced, abstract concepts or reasoning processes, and although I resonate in spirit with much of it, I have not wanted to take it as an assumption here. Rather, I want to see what positions are suggested independently by the case studies with regard to reasoning processes in science. Thus it was not just assumed from the outset here that L1 processes involve modal imagery and are grounded; hence the attention to Nersessian’s, Trickett’s and my own findings that support that. And although much of the research on grounded cognition has focused on the nature of *knowledge concepts* used in everyday conversation or activities, e.g., Barsalou (1999, 2009), the focus here has been on proposing that expert *scientific reasoning* that is capable of generating novel scientific models and inferences, can be grounded. One can also ask whether L2 or higher processes can be grounded, and this question will be examined in the discussion.

### Use of External Diagrams to Support Imagery

A broader related issue of situated or extended cognition is the role of external spatial representations, such as diagrams. Larkin and Simon (1987) described several possible advantages for using diagrams during problem solving, including supporting perceptual recognition, inferences, and order of search clues. They also speculated that these might also be advantages of internal imagery representations. All three book authors plus Trickett and Trafton (2007) hypothesized that diagrams can be an external support for modeling.

Darden (1991) included “Introduce and Manipulate a Symbolic Notation,” including diagrams, as a model generation strategy. Nersessian (1992, 2008) noted that Maxwell’s papers contained key diagrams, and remarkably, that his readers were instructed by him as to how to mentally animate them. Diagrams do not show animation and sometimes other features directly, and for S2 I have inferred that internal imagery was used, when evidenced by related action gestures animating a diagram like that of the hexagonal coil (protocol section 4), or by the subject’s speaking of relevant invisible features in a diagram. These both also indicate that the diagrams were insufficient on their own for representing certain relevant features.

Hegarty et al. (2003) studied students learning from diagrams about a device and found that learning was enhanced by asking them to make predictions about the behavior of its components, suggesting the importance of mentally animating static diagrams. In Trickett and Trafton (2007), spatial diagrams of observation patterns were provided as the main data sources for scientists to work from, and they detected scientists’ mental simulations of models, many of which were mentally projected onto the presented diagrams. Comparing via an internal simulation placed over an external image, is one species of what Trafton et al. (2002) and Clement (2004, 2008, 2009a,2018) termed an ‘alignment,’ or ‘overlay simulation,’ respectively, and since it is a possible method for evaluating an imagistic fit between an analogy and a target, or between a theoretical model and observations, it represents a very interesting form of extended cognition (cf. Vankov and Kokinov, 2011). See additional work on diagrams in, e.g., Hegarty et al. (2003), Gooding (2006), Craver and Darden (2013), Tversky (2015), Bechtel (2017), and Chandrasekharan and Nersessian (2017).

### Summary: The Framework as a Theoretical, Field-General Hypothesis Based on a Record of Exemplar Types

Regarding theoretical aspects of the framework, so far an attempt has been made to consolidate processes (strategies) and build on the GEM cycle identified by the three authors, introduce explicit levels in Figure 6, hypothesize greater size and time scales at higher levels, interpret the connections between levels as subprocess relations, move some larger processes the authors identified into a fourth Level 4, and hypothesize two other types of cycles there. I have also attempted to clarify the distinction between simulation and transformation processes at Level 1, and argue that these processes can be imagistic and grounded in the perceptual-motor systems, on the basis of think-aloud and neurological studies. The framework may also be viewed as a working typology of heuristics by different levels.

The hypotheses above were formed under many important constraints. Figure 6 partly serves as a record of 24 functionally diverse processes consolidated from the case studies in that each item in it corresponds to a process type derived from the books, shown in Tables 1–4^4^. The vertical and slanted arrows in Figure 6 also correspond to the types of L3–L2–L1 connections between processes that were indicated one or more times in Tables 1–3. Thus the framework is constrained for now by showing only the processes and vertical connections documented in the tables, plus the GEM cycle pattern there, and those framework components and structure have a basis in that sense. But the framework is expandable. Figure 6 suggests that most of the heuristic processes are field-general rather than field-specific. This complements the significant heuristics identified in Langley et al. (1987), many of which were field-specific or topic-specific (though some may be generalizable).

## Discussion

As a first order model, the framework in Figure 6 is a highly macroscopic functional sketch that includes many gray boxes with processes hidden underneath them that cannot be unpacked here, e.g. myriad strategies for experimental design hidden underneath the process “Take Features Inferred from Model and Ask if they Predict New Evaluatory Observations”. All three book authors agreed that the individual processes shown are heuristic and unreliable in the sense that individually they may be ‘inconsistently very useful’ but not guaranteed to work. However, since the case studies documented impressive achievements of creative model construction, one should ask, what are the strengths of the framework that may help us explain how a collection of such individually unreliable processes were able to overcome the significant challenges and pitfalls in creating a successful new theoretical model? Building from the case studies, I hypothesize these strengths below under the three objectives for the paper.

### Objective 1: Identifying a Set of Heuristic Reasoning Processes Used by Scientists During Creative Model Construction

A first result is that scientists use a considerable number of modeling processes–more than one might think– (at least the 24 indicated in Figure 6, with more in the books), complementing other studies that go into depth on a few heuristic processes. Most were field-general in appearing in more than one study. A major advantage for science is having multiple processes to try, important since heuristics can fail to work. For example, the framework presents a broader view than blind variation or prior concept combination alone being fundamental for creativity: e.g. combinations can arise from repeating the “Apply Schema to Model” or “Analogy” processes at L2; and non-blind variations can arise from “Altering Model Elements within Constraints” at L2 (cf. Chan and Schunn, 2015a, who provide evidence for the advantages of conducting improvement cycles in addition to conceptual combinations in product design).

### Objective 2: Asking Whether and How These Processes Are Organized and to What Advantage, if Any

#### Types and Degree of Organizational Structure

For simplicity and transparency I will describe the functioning of the framework here as if it was residing in one person with ordinary cognitive resources, and with the understanding that any real individual, e.g. S2 or Maxwell, might use only a portion of the processes, although teams might use a larger portion collectively. Is the above collection simply an unorganized ‘bag of tricks’ to try in any order? With 23 heuristics to choose from at each juncture, adding organization would reduce the size of the search space. The framework shows an hypothesized organization structure that does this and guides: including partial serial ordering, cycles, and a hierarchy (partially ordered set) of subprocess relations between processes at different levels, in contrast to the unorganized collection envisioned by Feyerabend (1975).

In particular, the model construction cycle at Level 3 was identified as a central organizing process by all three authors. Complex model construction in science is a hard, ill-structured problem, and such cycles may have two large advantages: to break the problem into parts via step-wise improvements; and to make possible recovering from faulty models via evaluation and modification. The GEM cycle also organizes the large number of heuristics at L2 in that each phase of the modeling cycle at L3 accesses a select subset of processes at L2. And Model Evaluation utilizes a set at L2 on the right in Figure 6 that is separate from the constructive processes on the left. (I don’t expect the last finding to necessarily hold perfectly true in adding future exemplars but it does suggest a distinct tendency.) Also at Level 4 the framework includes two other kinds of larger improvement cycles in addition to the GEM cycle that can break the problem into parts: increasing the degree of model precision incrementally, and moving to other ‘levels of explanation.’

In the above ways, the framework in Figure 6 is not ‘anarchistic’– it does have some organized structure that can break the problem in parts, recover from faulty models, guide an investigator and reduce the search space. But it is not fully algorithmic, in part because there are multiple unordered subprocesses involved. This places the framework as much more organized than Feyerabend’s anarchistic description, but much less algorithmic than Langley et al.’s (1987) computational descriptions of heuristics.

#### Balancing Divergent and Convergent Processes for Creativity

Based on the case studies, one can also hypothesize that the framework’s structure fosters a delicate balance between divergent (idea generating) and convergent (idea selecting, criticizing, or winnowing) processes. For example, one wants to be able to generate enough ideas to hit on the root of a successful model but also winnow enough to avoid being swamped by too many models or ending with a faulty one. Chan and Schunn (2015a) provide an informative review of various approaches to sequencing divergent and convergent processes, including: the use of divergent processes early, then convergent processes later (e.g., Amabile, 1983; Finke et al., 1996); multiple divergent/convergent cycles (e.g., Jin and Chusilp, 2006; Herring et al., 2009); or cycles with increasing degrees of convergence later (e.g., Goel and Pirolli, 1992; Ball et al., 1997; Clement, 2008; Nersessian, 2008; Chan and Schunn, 2015b).

Here, the central GEM cycle sequence can be seen as orchestrating a repeated alternation between divergent processes, on the left in Figure 6 at L3 and L2 and convergent processes on the right, as an important feature. Evaluations on the right are convergent in identifying faults and recognizing constraints on the model and target, or confirming or discrediting models. However, the three book authors agreed that the divergent L2 processes on the left owed part of their effectiveness to: (1) being used while heeding constraints accumulated from initial conditions and/or previous model evaluations; and to (2) aiming at specific faults during model modifications, which would leave the processes on the left still divergent but less so. This greatly increases the chance that the modification will be an improvement, vs. a random or blind modification (Clement, 1989; cf. Perkins, 1994).

The GEM cycle is thereby more powerful or ‘intelligent’ than the blind variation and test strategy described by Campbell (1960). And the cycle gradually becomes more convergent as constraints accumulate. However, the latter processes can involve critical difficulties, e.g., working memory limitations for multiple constraints, idea fixations on faulty models, or failures to access relevant knowledge, and I am not claiming that such model modifications can be done easily or via a guaranteed algorithm. They are still abductive conjectures needing evaluation, although they can be an educated conjecture. And some of the processes can involve associations/activations that can be spontaneous, such as finding a relevant schema or analogy (an additional way in which the framework is non-algorithmic.) In going beyond Campbell’s (1960) evolutionary theory of creativity as blind variation and selective retention, Simonton (2016, 2018) describes a broadly encompassing model of personal creativity, and argues that considered modifications can be anywhere between totally blind and totally sighted, falling on a spectrum of degrees of sightedness. The features above would put the framework somewhere in the middle, and moving gradually toward the sighted end as constraints accumulate.

Lest the incremental GEM cycle be taken as ruling out any possibility of conceptual reorganization or even revolution in science (e.g., for a coherent but faulty theory), it is still possible for the GEM cycle to get ‘stuck,’ due to the critical difficulties above. A long protocol section (referenced but not included in section 3, Table 2) was highlighted in Clement (2008) where S2 was frustrated and fixated on the faulty ‘bending’ model. S2 then appeared to suspend constraints and brainstorm more divergently, which led to his torsion insight. This suggests adding to the framework the capability of increasing the degree of divergence in such cases (cf. Chan and Schunn, 2015a; Hommel and Wiers, 2017; Mekern et al., 2019). Because the impasse was eventually followed by a sudden conceptual reorganization, then continuing improvement cycles, I described the overall trajectory as ‘punctuated model evolution’ (cf. Gould and Eldredge, 1977) rather than only a smooth series of small changes.

One can hypothesize how the framework’s various types of structural organization could enable an important but difficult balance between divergent and convergent processes. First, the guiding serial and subprocess relations in Figure 6 (inferred from Tables 1–3), can help reduce the number of strategy choices at each juncture. Secondly, without multiple specialized process options for model generation and modification on the left in Figure 6 at L3 and L2, and the possibility of increasing divergence when needed, one might not have enough divergent options to generate enough trial ideas or overcome idea fixations on faulty models. Scientists were also described as using less constrained followed by more constrained model modifications, allowing for wide divergence followed by fine tuning. Thirdly, processes on the right in Figure 6 were seen to include repeated evaluations, accumulating constraints, and multiple processes at L2 specialized for evaluation. These processes were seen to be convergent enough to constrain and prevent excessive divergence, eliminate faulty model ideas in play, and identify other faults that in turn motivated targeted improvements toward a viable model. Thus, there are many ways that various types of framework structure described in this section can be hypothesized to be advantageous for successful model construction.

### Objective 3. Can Scientific Reasoning Be Grounded? to What Advantage, if Any?

#### Level 1 Processes Are Grounded When Imagistic

Nersessian’s book and my own focused on two L1 processes, novel Mental Simulations for inferring the results of actions by a system, and Structural Transformations for changes a scientist makes in the structure of a system, concluding that they were often imagistic. Neurological reviews were cited providing strong evidence that both kinesthetic and visual imagery are grounded in the sense of using brain regions that are part of the perceptual and motor systems, implying that the two L1 processes are grounded when they utilize imagery.

#### How Might Grounded Processes at L1 Be Advantageous?

I will hypothesize that the L1 processes: 1. provide alternative resources for reasoning that can increase the number of creative options available to the modeler; 2. may have more specific advantages for some purposes. Alternatives to deduction or verbal combinations as resources for reasoning were indicated by the dotted boxes at Level 2 in Figure 6 indicating that, notably, there were connections for not just a few but for ten L2 heuristic processes being implemented by imagistic L1 processes. Each of those connections marks a way that grounded L1 processes were not just used on their own, but were seen as underpinning the operation of other ‘larger’ heuristics.

On the other hand there are limitations of imagistic processes (Hegarty, 2004). These include that they may be: primarily limited to qualitative, ordinal, or very small number relationships; sometimes faulty or misapplied for scientific purposes (e.g., in the extensive literature on science and probability misconceptions); and subject to capacity limitations of imagery systems, although in other cases imagery can represent a large amount of spatial information more efficiently than linguistic symbols.

Secondly, despite these limitations, Schwartz and Heiser (2006) review evidence for specific advantages of imagery representations for learning, including the ability to represent interconnected spatial features efficiently, and to recognize emergent forms when combining images (Finke, 1990).

Transformations and Simulations at L1 are each hypothesized here to have more specific beneficial divergent and convergent functions, with each process serving L2 parents on both the left and right sides in Figure 6. For novel Imagistic Simulations: (1) In a convergent model evaluation role, they can be a very efficient and major alternative to sequences of deductions for generating inferences from a qualitative model, to generate explanations and compare with observations, as proposed in Trickett and Trafton (2007), Clement (2008), Nersessian (2008), and Trickett et al. (2009). For example, S2’s simulations in sections 7 and 8 of Table 2 produced confident inferences very rapidly. (2) Extending this inference generating role, in cases where the output result of one simulation can be the input state of another, simulations can participate in modeling causal chains efficiently (cf. Metz, 1998; Schwartz and Black, 1999; Hegarty et al., 2003; Hegarty, 2004; Clement, 2008) hypothesized here to provide satisfying explanations. (3) In a divergent role that can lead to model generation or modification, a simulation can spontaneously activate dormant but relevant knowledge schemas imagistically, such as the twisting and torsion schemas in S2’s insight (episode 5, Table 2).

One can also hypothesize strengths for Imagistic Structural Transformations: (1) They can be a major subprocess for building an imageable model under constraints by combining elements. Diagrams or physical models can help here as well. Having built on work by Franklin, Pauling and others, Watson (1970, p. 194) describes his insight in using cardboard models of bases, where he stopped going “back to my like-with-like prejudices” and “began shifting the bases in and out of various other pairing possibilities” which led to a key insight on how they could fit spatially into a double helix. This exemplifies trying transformations under constraints to build a model (and breaking out of an idea fixation). Presumably this must have involved internally imagining multiple chemical bonds of different types to and between the bases. (2) Similarly, in both Clement (2008) and Nersessian (2008) it was argued that Imagistic Transformations may be an important method for modifications targeted to remedy faults or gaps under multiple spatial and physical constraints, e.g., Maxwell’s introduction of tiny vortices (like idle wheels) to remove ‘friction’ between vortices. (3) Alternatively, when playful, with fewer constraints, Imagistic Structural Transformations may provide more divergent ideas (Finke, 1990). Research on structural transformations is very sparse and sorely needed (but see Wertheimer, 1959; Clement, 2008, 2009a; Shegeva and Goel, 2021).

The above strengths of imagistic L1 processes may support the difficult critical needs identified earlier of (1) evaluating and modifying a model to gather and satisfy accumulated constraints, plus (2) finding alternative paths to generate divergent model ideas and to break out of idea fixations. This provides a basis for hypothesizing that the grounded, imagistic processes at L1 each have significant value for scientific reasoning in both divergent and convergent roles, as seen in Figure 6.

#### Can Level 2 Processes Be Grounded?

Earlier I argued that the two L1 processes can be grounded, defined in the sense of utilizing the perceptual and/or motor systems in the brain componentially, and that L2 processes in the dotted boxes in Figure 6 utilized a grounded L1 process as an important subprocess. In other words many L2 processes can utilize subprocesses at L1 that utilize imagery. One can ask whether those L2 processes are thereby grounded. If one accepts the idea that a process that utilizes an important subprocess that utilizes the perceptual or motor systems is grounded because it too is utilizing those systems, then according to the above definition, the latter L2 processes can be grounded in this transitive, ‘componential’ sense. This view fits well with the S2 transcript which contains many examples of tight coupling between L1 and L2 processes, and imagery indicators occurring concurrently with them in sections 4, 5, 7a, 7b, 7c, 8, and 9. In some cases, action gestures can even depict L1 and L2 processes simultaneously in same gesture, such as in Episode 4, where the action gesture for bending wire to form a hexagon is an acting out of both the L2 process of altering a model and conducting an Imagistic Structural Transformation at L1. The tight coupling suggests that the L2 process is grounded, at the very least in the componential sense. This is one sense in which one can say that higher order, expert reasoning processes can be grounded.

Some might hesitate to speak of ‘componential grounding’ across levels because it might imply ‘grounding all the way up’ to Level 3 or beyond, and that may be true in some cases. However, there could still be cases where an L3 process was not grounded, either because: an L2 (process) was triggered spontaneously without being activated by an L3; L3 activated L2 but was not still active when L2 operated because the size of working memory was limited (Altmann and Trafton, 2002); or L2 operated via deduction. These complications suggest that many semantic, system architecture, and modeling issues can be raised in this area for future research.

The most common use of simulations by scientists in Trickett and Trafton’s (2007) protocols was to run a simulation of a newly hypothesized model and see if it explains a spatial map of the data. This corresponds to an important vertical implementation path in Figure 6: using Imagistic Simulation at L1 as a subprocess for ‘Inferring a Feature of a Model by Running It and Asking if it Explains Relevant Observations’ at L2, as part of ‘Evaluating a Model’ at L3. They observed that subjects often evaluated a mental model directly by running it in a mental simulation and aligning it with the spatial data display while staring at it, saying: “the process of alignment was primarily based on perception because of the visual–spatial nature of the scientists’ data.” Thus they provided evidence for such a closely coupled use of concurrent processes at L1 and L2 in their subjects, who came from a variety of scientific fields. This adds to the present argument that certain scientific reasoning processes at L2 can be grounded by being implemented at L1 via the perceptual/motor systems.

I am not arguing that these L2 heuristics always operate via imagistic processes; e.g., sometimes they may operate by deduction (Cellucci, 2020), and I have named the L2 processes in Figure 6 to allow for that. But the above findings provide initial grounds for adding to the framework that expert scientific reasoning processes, at least at L1 and many at L2, can be grounded by being implemented via imagistic processes utilizing the perceptual/motor systems.

### A Strong Coalition

In summary, the modeling processes framework in Figure 6 contains a consolidated set of important, mostly field-general reasoning processes used during episodes of innovative theory construction. Since its heuristic processes are only sometimes useful with uncertain utility individually, possible strengths of the framework have been proposed that may help explain how such processes can work together to produce successful and innovative scientific models. These include its wide range of processes to try, its flexible, organizational structure via cycles and hierarchical subprocess relations that provide partial guidance and allow it to balance sources of divergence and convergence for creativity, and the hypothesized benefits of grounded imagistic processes at L1. These strengths may begin to explain how the framework can function as a relatively powerful coalition of processes for constructing innovative scientific models even though each process has uncertain utility. By continuing to investigate how heuristic processes can be organized, we may add clarity to their purposes, structure, and functioning.

## Questions for Future Research

### Knowledge Representations

For reasons of focus this paper concentrates predominantly on reasoning processes. Knowledge representations for models, observations, cases, sources, and constraints have not been addressed except to say that imagery can be heavily involved, and I will briefly steer the reader toward questions raised and work by the three authors on this issue.

#### Representation for the Model Being Constructed

One possible direction for future research is the hypothesis that imagistic representations of a model could have distinct advantages for representing a number of accumulated, interacting spatial and physical constraints efficiently in a single representation (Clement, 2008; Nersessian, 2008). This should be added to the hypotheses in the previous section on possible advantages of the imagistic processes at Level 1. Carroll et al. (1980), cited in Reed (2016) provided some indirect support for the latter in finding that students who designed a business office by drawing diagrams satisfied more given constraints than students who worked on the manufacturing process and did not draw diagrams. The two groups were equally successful when both were instructed to use diagrams.

One could also explore connections to the nature of scientific *mechanisms* as knowledge representations (Machamer et al., 2000; Darden, 2006; Craver and Darden, 2013). Related to the concept of *models* used here, their work paints a detailed, abstract description of a theoretical mechanism as a system of parts that interact and generate systematic changes, and that underlie and explain observed behavior patterns in science. This includes the mechanism’s structure, functions, and activities, and sorting out how structures and functions are nested at different layers of a system, such as organs, tissues, and cells. None of the three books considered here focused on unpacking the concept of mechanism at the time of their writing.

#### Knowledge Sources for Model Construction

Nersessian (2008) reviewed research by Schwartz and Black (1986) and others indicating that depending on the task, subjects could use different types of knowledge sources to replace or complement mental simulations. For example, in predicting the behavior of gear trains, subjects might use a chain of mental simulations with accompanying tacit knowledge, but at other times could also use more formal knowledge of angles, viscosity, and gravity, as well as verbal rules about alternating rotation directions (cf. Stieff and Raje, 2010). Having analyzed Maxwell generating perhaps the most abstract scientific model of the 19th century, Nersessian (2008, p. 180) was acutely aware of the diversity of knowledge types that can provide constraints for modeling: “by means of linguistic, formulaic, and imagistic informational formats, including equations, texts, diagrams, pictures, maps, physical models, and kinesthetic and auditory experiences”.

In Clement (2008) I outlined how formal geometric, and algebraic knowledge schemas could be applied and added to the model of the spring, once a fully connected, runnable, qualitative model with ordinal (direction of change) causal relationships between elements in the spring had been constructed. It was hypothesized that this requires a high level of precision in imagining detailed features of the model and its actions as well as precision in using consistent, verbal and algebraic names for those essential features. Marghetis and Núñez (2013) reported the use of dynamic image schemas as metaphorical knowledge sources used by six pairs of expert mathematicians in formal proof discussions on the basis of their videotaped action gestures.

#### More Work Needed on Connections to Perceptual-Motor Schemas

It is also important to prevent the impression that reasoning via imagistic simulation can create new knowledge out of nothing without drawing on prior knowledge sources. The square spring model in episodes 7b-c in Table 2 appeared to be run by drawing on elemental perceptual-motor schemas for real bending and twisting actions on objects; it is hypothesized that those schemas were run there vicariously on sides of the square coil by generating dynamic imagery. Presumably such schemas ultimately build on the gradual development of basic ‘intuitive physics’ concepts from childhood (e.g., diSessa, 1985; Clement et al., 1989; Spelke, 2000; Feist, 2006; Wilkening and Cacchione, 2011; Carey, 2009). The square coil is not S2’s final model, but his running it serves as an example of a *compound* imagistic simulation in which there is both a utilization of old knowledge schemas and inventive reasoning by running a new and novel combination of those schemas, aligned spatially in a new way, to form a causal chain with new inferential consequences. As described in Clement (1994, 2008; Clement, 2009b), this can also include tapping previously unarticulated, implicit knowledge lying within such schemas in cases like episodes 7C and 8 in Table 2, and the use of spatio-physical reasoning. Imagistic Transformations and Simulations may ultimately derive their advantages from tapping into such natural perceptual-motor resources for tool-making within constraints and for anticipating how objects interact spatio-temporally, respectively. Future approaches will likely need to include the role of elemental action components such as anticipatory predictions from schemas in motor control theory (e.g., Schmidt, 1982; Grush, 2004; Barsalou, 2009; Kühn et al., 2011). On the other hand, since novices can harbor some schemas that are faulty or misapplied for scientific purposes, those can generate faulty simulations, also indicating the importance of one’s knowledge source schemas for successful modeling.

This leaves many questions open in need of study about the nature of knowledge representations being used in science, which may involve: connections between pre-symbolic and symbolic representations via pluralistic views of *knowledge representations* (e.g., Dove, 2009; Markman, 2012; Matheson and Barsalou, 2016), and hybrid architectures that integrate both (e.g., Harrison and Schunn, 2002; Helie and Sun, 2010; Blouw et al., 2016). Also central is examining the relationship between knowledge and reasoning processes in science (e.g., Klahr and Dunbar, 1988; Schunn and Klahr, 1995; Craver and Darden, 2013; Fugelsang and Mareschal, 2013).

### Toward a Revised, ‘Softer’ Reasoning Framework: Somewhat Less Controlled but More Divergent and Flexible

In future modeling the framework may need to be ‘softened,’ to account for other phenomena such as those in Clement (2008) that seem less controlled, e.g., the generation of multiple divergent ideas at ‘brainstorming’ points; ‘off task’ remindings, or the sudden new application of a knowledge schema. These could be explained by spontaneous spreading activations (perceptual or verbal) to knowledge schemas, as could S2’s Aha! in section 5 of Table 2 involving the sudden activation of twisting and torsion schemas from running the newly created hexagonal coil model.

But the latter insight also involves an *interruption* of the subject’s current reasoning process, as he suddenly switches from examining the bending model to evaluating the twisting idea. Combined with the problem of how unordered subprocesses in the framework are selected, these phenomena also imply less control over reasoning strategies and more flexibility than a more strictly programed hierarchy. One can speculate that to explain these phenomena, future versions of a modeling framework should include: (1) spreading activation of knowledge schemas; (2) parallel activation of alternative strategy units in the framework, with a number of sources influencing which strategy becomes activated most strongly and implemented. This would allow for competition resolution mechanisms for decisions between unordered substrategies, and opportunistic interruptions of a process (cf. flexible, hierarchical, competitive activation networks for actions in Norman and Shallice, 1986; Anderson, 1993; Thagard and Millgram, 1997; Cooper and Shallice, 2000; Hommel and Wiers, 2017). These features could be combined with the present framework in Figure 6 as properties of connections and strategy units that bias and incline but not always require the scientist to activate and use the subprocesses and sequences shown, in what one might term a ‘soft hierarchy.’ Allowing interruptions from spontaneous activations of schemas would add variation and divergence to idea generation in the framework, moving the framework somewhat further from the ‘sighted’ end of Simonton’s (2018) scale of degrees of sightedness. One could also include redistribution of activations after negative feedback from model evaluations (Ohlsson, 2011), plus process preconditions, utility and cost estimates, and bounded rationality limitations such as limited working memory resources for goals, plans, imagery, and verbal processing (Altmann and Trafton, 2002; Jilk et al., 2008). The collective features above could contribute to explaining the above phenomena indicating less control but greater flexibility.

Another implication, partially analogous to parallel activation across levels in Cooper and Shallice’s (2000) and Cooper’s (2019) multi-level action hierarchies, is that one could envision a connected subprocess string of three processes at L3, L2, and L1 (e.g., model modification via adding an element via an imagistic structural transformation in Figure 6), each staying activated in parallel to some degree during their operation, with the L3 and L2 processes each ‘monitoring’ the results from those below them for completion. All three processes might also be attending to an imageable model representation simultaneously. This would suggest the alternative possibility of a more direct form of ‘entrained’ parallel grounding of L2 and possibly L3 processes rather than only the serial form of componential grounding of ‘calling’ a grounded subprocess at L1. Indeed, in instances like episodes 7B-C in Table 2 the L1–L2–L3 relationship appears to be so simultaneous, tightly coupled, and entrained, and the dynamic perceptual-motor imagery appears to be so central to the L2 and L3 processes, that it could support referring to their ‘entrained grounding.’

These indicate some possible directions for future research. Because of the similar patterns in the three book studies and the Trickett and Trafton (2007) paper reviewed here, I have proposed the partial serial, cyclical, and hierarchical levels organization of the processes in the framework in Figure 6 as a first order model, while recognizing that similar patterns of connections in future models may take the form of activation contributions amidst bounded capacities. This may relate to those advocating pluralistic models of serial and parallel *processes* (e.g., Jilk et al., 2008; Trafton et al., 2013), with higher levels in a hierarchy involving more sequential control over longer periods, and lower levels involving faster, parallel processing of imagined perception and action.

## Limitations

1. Since the major sources here were case studies, one cannot claim to have a tested theory. However, the records in the case studies, provide a large number of significant constraints on hypothesis generation. One way case studies can be valuable is to acquire a toehold in developing a plausible theoretical sketch in the early stages of a field, especially in an area where qualitative mechanisms are sparse or unknown. The present highly macroscopic framework is intended as both a ‘field map’ or summary of species of reasoning processes used and as an initial functional sketch of the relationships between them. The framework is certainly oversimplified, is limited for now to excerpted processes from the case studies, and is meant to be expanded and improved. It is idealized because it consolidates reasoning processes from multiple examples of exemplary model construction.
2. One cannot claim evidence here for either conscious, or explicitly named processes by a subject, except in occasional places where they are described by subjects in the history or protocol records. Along these lines, Barsalou (2009) has theorized that grounded concepts need not always generate conscious imagery in order to be utilized. How much is conscious is an open question for future research.
3. Despite the evidence for imagery use reported here, it may be objected that *some* scientists do not report using much imagery in their work. A survey by Hadamard (1945) found great variation in mathematicians’ responses about whether they were conscious of using imagery, whereas Einstein and many others were quite explicit about its central importance (cf. Marghetis and Núñez, 2013).
4. Here experts were reasoning about complex unobservables while inventing sophisticated scientific models. But a number of the reasoning processes may be developmentally connected to everyday versions of reasoning, such as mental simulation, analogy, model generation, and evaluation via observations (Lehrer and Schauble, 2015). For example, in one of the few collections of case studies of creative thinkers Weisberg (2006) argues that experts used many everyday thinking processes; Lombrozo (2019) examines the role of thought experiments in everyday learning; Metz (1998) and Koslowski (2013) argue that children can do several types of scientific reasoning; and Sternberg (2006) argues that processes used during normal science can be creative. An implication is that work on frameworks may inform socio-cognitive approaches that are badly needed to teach scientific thinking skills (e.g. Osborne et al., 2004; Schwarz et al., 2009; Wiser and Smith, 2009; Windschitl et al., 2012; Lattery, 2016; Bielik et al., 2021), including grounded (Stephens and Clement, 2010; Newcombe, 2013; Price et al., 2017; Alibali and Nathan, 2018; Ramey et al., 2020; Mathayas et al., 2021) and hierarchical approaches (Schoenfeld, 1998; Williams and Clement, 2015; Nunez-Oviedo and Clement, 2019).
5. For the hypothesized correlation of longer time scales to higher process levels, process instances are going to be variable, and sometimes thrown off by getting stuck or by process interruptions. One may only be able to hypothesize for example, that on average in large samples, processes at L2 are shorter than those at L3. And all will vary with expertise and the difficulty of the problem, so the hypothesis is for relative durations between levels within those parameters. The latter is compatible with our learning from both large scale modeling in real science and smaller scale modeling in laboratory interviews.

## Conclusion

In this study, 24 heuristic processes for scientific model construction were consolidated from three detailed case study volumes, and most appeared to be field-general processes. However, none were guaranteed to work, and without any ordering structure, trying to use them could still encounter too large a search space for finding a successful model. Each of the three main authors identified a central model generation, evaluation, and modification (GEM) cycle utilizing constraints that provided some initial structure. It was seen to allow scientists to build a model incrementally, and often to recover from faulty or incomplete models.

From an analysis of patterns of use in the consolidated case studies, additional structure was proposed in the modeling processes framework in Figure 6 as a process hierarchy of four nested levels operating at different size and time scales, with some processes serving as subprocesses for other ‘larger’ processes. It depicts a partial organization of serial, cyclical, and hierarchical relations between processes, but with many unordered subprocesses. The resulting framework has an intermediate degree of organization that is neither anarchistic, nor fully algorithmic. A general long-range challenge here is to understand how reasoning processes can be organized and interconnected at different levels.

Since the processes are heuristic and only sometimes useful individually, strengths of the framework’s organization were hypothesized to explain how the processes can produce successful and innovative scientific models within the bounded resources of a cognitive system. The GEM cycle was described as alternating access to divergent and convergent subprocesses in order to achieve a difficult balance between them, typically with increasingly constrained modifications to the model occurring on each cycle. Other issues discussed included: consciously increasing divergence to break out of idea fixations, leading to occasional insights and producing a ‘punctuated evolution’ trajectory of investigation; and ‘softening’ future versions of the framework to be more flexible by viewing the links between processes as influencing their activation levels rather than indicating an inflexible procedure.

Findings from Nersessian (2008) and from think-aloud video studies of qualitative modeling (Clement, 1994, 2008, 2009b; Trafton et al., 2002; Trickett and Trafton, 2007) suggested that scientists do not just rely on logical symbol manipulations, but can utilize imagistic simulation and transformation processes. Where there is evidence these expert cognitive processes are imagistic, it can be argued from previous neurological findings that they are grounded in utilizing the perceptual or motor systems in the brain. Imagistic transformation and simulation processes were hypothesized to be particularly advantageous for model modification and inference making from running a model, respectively, under multiple spatial and physical constraints, among other benefits such as imagistically activating dormant but relevant knowledge schemas. Long range challenges here are to understand how higher order reasoning processes can be integrated with or underpinned by imagistic reasoning processes –or more generally, how to describe relationships between more prolonged, quasi-procedural, serial and cyclical processes and fast, parallel, grounded processes derived from those one uses to actually perceive and manipulate objects.

Altogether the consolidation attempted to: (1) identify a diverse set of heuristic processes for modeling, each one of uncertain utility in being only sometimes useful and not guaranteed to work; (2) infer some ways in which they can be organized and grounded; and (3) hypothesize some possible strengths of that organization. These were motivated by the ongoing challenge to explain how these processes can form a strong coalition to achieve innovative scientific model construction, even though each process has uncertain utility.

## Data Availability Statement

The transcript data supporting the conclusions of this article will be made available by request, without undue reservation.

## Ethics Statement

The studies involving human participants were reviewed and approved by the Institutional Review Board, Research and Engagement, University of Massachusetts Amherst. The participants provided their written informed consent to participate in this study.

## Author Contributions

The author confirms being the sole contributor of this work and has approved it for publication.

## Conflict of Interest

The author declares that the research was conducted in the absence of any commercial or financial relationships that could be construed as a potential conflict of interest.

## Publisher’s Note

All claims expressed in this article are solely those of the authors and do not necessarily represent those of their affiliated organizations, or those of the publisher, the editors and the reviewers. Any product that may be evaluated in this article, or claim that may be made by its manufacturer, is not guaranteed or endorsed by the publisher.

## Footnotes

1 Some of the other distinct heuristic strategies at level L2 identified in Darden (1991) that I do not have room to exemplify here are: Under Model Generation: Introduce and manipulate a symbolic representation, Invoke a theory type, Begin with vague idea and successively refine. Under Model Evaluation: Use other Refined Criteria such as clarity, scope and generality, fruitfulness, quantitative when possible. Under Model Modification: Localize problem, Generalize or specialize, Delineate (differentiate concepts) and change.

2 Some other distinct strategies identified in Clement (2008) that I do not have room to discuss are: Within Level L2- Under Model Generation: Partitioning, several types of Analogy; Under Model Evaluation: Symmetry argument; Under Model Modification: Concept differentiation, Aligning the model spatially to target phenomenon, Generalization, Transfer of Runnability to model from source schemas; Within Level L1- Compound simulations, Imagery enhancement, Overlay simulations, Conserving transformations.

3 Some other distinct strategies identified in Nersessian (2008, p. 184) are: Under Model Generation: Generalization; Evaluation: Goodness of fit of the model to: Constraints, Mathematical Implications, and Explanatory Power; Modification takes place according to the enhanced understanding of target, source, and model constraints.

4 This includes the assignment of L1 processes to the six rows of Table 3 described earlier.
