# Supplementary material for: Multiple Levels of Heuristic Reasoning Processes in Scientific Model Construction
Source: Front Psychol. 2022 May 10;13:750713. doi: 10.3389/fpsyg.2022.750713 (PMC9127582; doi:10.3389/fpsyg.2022.750713)
Supplement: Supplementary file 1 [file Presentation_1.pdf]

Supplementary Material

Appendix 1- Introduction to Concepts of Torque and Torsion

The concepts of torque and torsion can be introduced via Figure S1 below. For torque, if we ignore segment *hb* we can think of segment *ab* as a pipe and segment *ga* as a pipe wrench that we are using to turn the pipe clockwise, so that the threaded pipe is forced to go into a tight, threaded socket at *b*. Then **torque** can be thought of roughly as the 'twisting force' applied by the wrench to the end of the pipe at *a* to turn it. The torque will be greater in proportion to the length of the wrench, *r*, i.e. longer wrenches provide more leverage and more 'twisting force'. (When the force *F* is perpendicular to *r*, the amount of torque *T* applied to the end of the pipe is equal to the applied force *F* times the length of the wrench *r*.)

To introduce **torsion**, we need a different scenario. Imagine that *ab* is a steel rod only 1/8" thick (e.g. made of coat-hanger wire) with the bent end *bh* clamped in a vice so that the far end of the rod at *b* cannot turn. Then if we firmly attach a wrench *ga* to the near end of the rod at *a*, applying the same force will twist the end of the rod at *a* and introduce a twisting deformation into the thin metal rod along its length, since the end at *b* cannot turn. The near end at *a* will turn through the angle ß shown in Figure S1 (called the angular displacement at *a*, or, informally, the total amount of twist in the rod) and stop. **Torsion** refers to such an action that creates a twisting deformation in a material. If the rod is made of resilient metal, that deformation in turn will make the rod want to spring back to its original shape. It will be elastic, meaning that if we remove the force *F*, the metal in the rod will untwist and spring back to its original orientation where ß was zero.

If the rod *l* is twice as long, but *r* and *F* are the same, the angle ß will double when the force is applied. That is because the 'twisting force' (torque) will be the same as before, but there will be twice as much metal that can deform under that same torque, producing twice the total twist ß in the rod. S2 refers to this effect qualitatively in section 7c of Table 2.

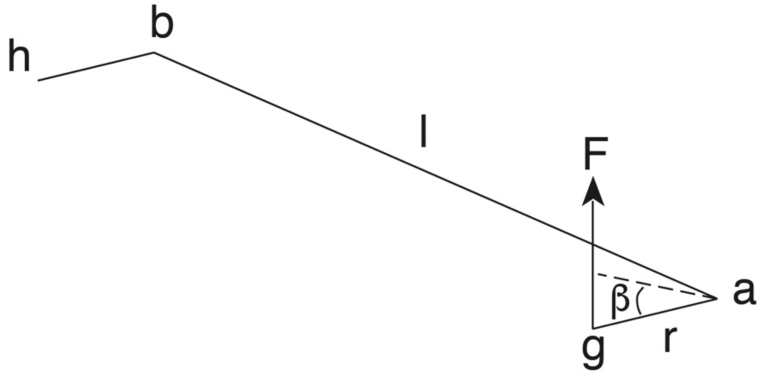

Figure S1. Torque and torsion
